# Tomato Fruits Show Wide Phenomic Diversity but Fruit Developmental Genes Show Low Genomic Diversity

**DOI:** 10.1371/journal.pone.0152907

**Published:** 2016-04-14

**Authors:** Vijee Mohan, Soni Gupta, Sherinmol Thomas, Hanjabam Mickey, Chaitanya Charakana, Vineeta Singh Chauhan, Kapil Sharma, Rakesh Kumar, Kamal Tyagi, Supriya Sarma, Suresh Kumar Gupta, Himabindu Vasuki Kilambi, Sapana Nongmaithem, Alka Kumari, Prateek Gupta, Yellamaraju Sreelakshmi, Rameshwar Sharma

**Affiliations:** Repository of Tomato Genomics Resources, Department of Plant Sciences, University of Hyderabad, Hyderabad, India; Universita degli Studi di Siena, ITALY

## Abstract

Domestication of tomato has resulted in large diversity in fruit phenotypes. An intensive phenotyping of 127 tomato accessions from 20 countries revealed extensive morphological diversity in fruit traits. The diversity in fruit traits clustered the accessions into nine classes and identified certain promising lines having desirable traits pertaining to total soluble salts (TSS), carotenoids, ripening index, weight and shape. Factor analysis of the morphometric data from Tomato Analyzer showed that the fruit shape is a complex trait shared by several factors. The 100% variance between round and flat fruit shapes was explained by one discriminant function having a canonical correlation of 0.874 by stepwise discriminant analysis. A set of 10 genes (*ACS2*, *COP1*, *CYC-B*, *RIN*, *MSH2*, *NAC-NOR*, *PHOT1*, *PHYA*, *PHYB* and *PSY1*) involved in various plant developmental processes were screened for SNP polymorphism by EcoTILLING. The genetic diversity in these genes revealed a total of 36 non-synonymous and 18 synonymous changes leading to the identification of 28 haplotypes. The average frequency of polymorphism across the genes was 0.038/Kb. Significant negative Tajima’D statistic in two of the genes, *ACS2* and *PHOT1* indicated the presence of rare alleles in low frequency. Our study indicates that while there is low polymorphic diversity in the genes regulating plant development, the population shows wider phenotype diversity. Nonetheless, morphological and genetic diversity of the present collection can be further exploited as potential resources in future.

## Introduction

Tomato (*Solanum lycopersicum*) is one of the major vegetable crops cultivated globally and ranks second in production after potato (*Solanum tuberosum*) worldwide [1]. Tomatoes are rich in sugars and free acids, which are the key components determining taste, whereas, volatile organic compounds determine the flavor [2]. Tomatoes contain essential as well as beneficial components like carbohydrates, fiber, minerals, protein, fat, glycoalkaloids, phytosterols etc. [3]. Several essential vitamins like vitamin A, vitamin C, vitamin E, folic acid and several water-soluble vitamins are also present in tomato [4]. The ripened tomato fruits are deep red colored owing to the accumulation of lycopene which constitutes more than 80% of total carotenoids. Lycopene is known to be a powerful antioxidant, anti-cancerous agent and enables protection against neurodegenerative diseases [5]. Additionally, other minor carotenoids such as *β*-carotene, *γ*-carotene, phytoene also contribute to the antioxidant property of tomato. Other than antioxidant property, *β*- and *γ*-carotenes also have pro-vitamin A activity. Tomato also has flavonoids like chlorogenic acid, rutin, and plastoquinones, tocopherols that have antioxidant properties [4].

Given the problem of malnutrition in developing nations, tomato is considered as one of the potential crops for increasing the yield as well enhancing the nutraceuticals levels. Owing to simple diploid genetics and small genome size (12 chromosomes with 950 Mb size), rapid propagation, short life cycle, susceptibility to diseases, and climacteric fruit ripening, tomato is considered as a model crop for functional genomics studies [6]. The nearly complete tomato genome has been published [7] and supplemented by sequences of a large number of heirlooms, landraces and hybrid cultivars [8, 9]. Sol Genomics Network (SGN) [10, 11] acts as an open access repository of information about the genome sequences, genes, pathways, phenotypes, maps, markers, mapping populations, etc. for tomato and other Solanaceous species and has links to available bioinformatics resources. The structural variation browser for tomato and its wild relatives is also available [12].

Traditionally, tomato improvement has been carried out by classical breeding approaches by introgressing genes from the wild relatives. However, the above approaches require longer time to modify a trait and do not entail information about the causative genes. The availability of genome sequences now allows detection of mutations/SNP in a given gene. A strong genotype-phenotype linkage can be used to select the desired alleles to improve the trait related to gene function. Using Targeting Induced Local Lesions In Genomes (TILLING) the mutations in a specific gene can also be selected for crop improvement. In several crop species, repositories of genomic DNA and seeds have been generated as part of TILLING efforts and are accessible to potential users to seek mutations in a specific gene [13–16]. The protocols used for TILLING has also been applied to detect variations in the genomic DNA of natural accessions/cultivars and termed as EcoTILLING. In natural populations, EcoTILLING can efficiently detect the Single Nucleotide Polymorphisms (SNPs) and small InDels which are the most common form of nucleotide variations [17].

The search for natural variation in specific genes in crop species has led to the identification of several accessions with improved traits. In melon, EcoTILLING of eukaryotic translation initiation factor 4E (*eIF4E*) led to identification of a new allele that imparted resistance to Melon necrotic spot virus (MNSV) [18]. Similarly, identification of SNPs in *eIF4E* in *Capsicum* accessions led to identification of five new *eIF4E* variants that were related to Potato virus Y-resistance responses [19]. In rice, a combination of EcoTILLING in promoter sequences of 24 transcription factors and association with drought tolerance led to identification of three and five genes that were associated with the drought tolerance index and level respectively [20]. Likewise in *Brassica rapa*, using EcoTILLING, [21] identified three SNPs in two *FAE1* paralogs that were associated with low erucic acid (LEA) content that can be exploited for LEA breeding. The utility of EcoTILLING can be further enhanced by combining it with the phenotyping of natural accessions. A deeply phenotyped crop population with a collection of genomic DNA can serve as a resource for the screening of natural polymorphism in genes related to a given trait to seek genotype-phenotype association. The stronger SNPs-phenotype associations for different traits can be used for developing new cultivars through breeding. A genome-wide association analysis involving 163 accessions of red-fruited species of tomato clade delineated 44 loci associated with 19 metabolite traits providing putative candidate genes for crop improvement [22].

In the present study, we phenotyped a large number of tomato accessions with special emphasis on fruit morphological and biochemical parameters. The phenotypic diversity of the population was assessed utilizing high throughput digital data collection tools such as PDA [23] and Tomato Analyzer [24] in combination with the assessment of SNPs by a reverse genetic screen, EcoTILLING. Statistical analysis of the morphometric data by TA showed fruit shape to be a complex trait. The accessions with desirable traits were identified in the population. Our study also reveals that the fruit development is less significantly correlated with the SNPs in analyzed genes implying for a more complex regulation of this trait.

## Materials and Methods

### Tomato accessions

Seeds of tomato accessions were obtained from different resources viz. National Bureau of Plant Genetic Resources (NBPGR, New Delhi, India), Indian Institute of Vegetable Research (IIVR, Varanasi, India) and Tomato Genetic Resource Centre (TGRC, California, USA). An Indian cultivar of *Solanum lycopersicum*, Arka Vikas (Sel 22), was used as a reference variety. The accessions were collected from various sources in India on a random basis except for accessions obtained from TGRC.

### Seed germination and plant growth

Ten seeds of each accession were surface sterilized with 4% (v/v) sodium hypochlorite solution for 5–10 min and washed thoroughly under running tap water. The seeds were sown on wet blotting sheets in petriplates and germinated in darkness at 25±2°C. Individual germinated seeds were transferred to 96-well seed germination trays with perforated bottoms. The wells were filled with coconut peat (Sri Balaji Agro Services, Madanapalli, India) and trays were placed in larger trays filled with 2 cm water. The seedlings were grown at 25±2°C under coolwhite fluorescent lights (100 μmol/m2/sec) for 10–15 days. After the emergence of secondary leaves, the cotyledons were collected from each accession for DNA extraction. After that the seedling trays were transferred to the green house, and at 5–6 leaf stage, the plantlets were transferred to larger pots with drip irrigation in the open field containing soil-manure mixture with 30% sand. Six seedlings of each accession were transferred to pots with two seedlings per pot.

### Phenotyping of plants

Each accession was tagged with a unique plant-ID barcode label as described earlier in [23]. At different developmental stages of the plant, 16 major phenotype categories [25] were recorded using a hand-held Personal Digital Assistant (PDA) equipped with barcode scanner and customized software, PHENOME, for large-scale phenotypic data collection [23]. The phenotypic variations were recorded using a digital camera. All recorded phenotype data and images were transferred to a central PC for further analysis.

### Morphological and biochemical analyses

At the anthesis, flowers were tagged, and fruits were collected at 40 days post-anthesis (DPA) from each accession for measuring morphological and biochemical analysis. The fruits from most accessions at 40 DPA were at the red ripe stage except non-ripening mutants. After harvest, the fruit weight and diameter was measured. The fruit diameter was measured both in vertical and horizontal axis using a digital Vernier caliper (Mitutoyo Absolute Digital Caliper, Japan). Thereafter fruits were homogenized using mortar and pestle, filtered through a sieve and the pH of the homogenate was measured with a pH meter. A 300 μL aliquot of the homogenate was used for measuring the TSS using a refractometer (Atago ® Pocket Refractometer PAL-1, Japan) calibrated with 300 μL milliQ water.

#### Carotenoid estimation

Total carotenoids were extracted from tomato fruits using the protocol described by Sadler et al. [26] with few modifications. All the steps in the extraction and estimation were carried out under subdued ambient light condition. About 0.5 g of red ripe fruit tissue (pericarp and mesocarp) was homogenized with mortar and pestle, and the homogenate was filtered through a sieve to remove debris. A 50 μL aliquot of homogenate was transferred to a 2.0 mL Eppendorf tube and 1.25 mL of solvent mixture (Hexane: Acetone: Absolute Alcohol in 2:1:1, v/v/v ratio) was added. The tubes were thoroughly vortexed and stored on ice. To the mixture 195 μL distilled water was added and after mixing, tubes were centrifuged at 10,000 rpm at 4°C for 10 min. 1 mL of the supernatant was withdrawn for carotenoids estimation.

The total carotenoid content was estimated using a method based on mean absorption wavelength (451 nm) and mean absorption coefficients [27]. A451nm of the supernatant was measured, and amount of total carotenoid content in μg/g fresh weight (FW) tissue was calculated. The carotenoid content was estimated by using the formula described by Rodriguez-Amaya and Kimura [28].

$$\text{Total carotenoid content}\;\left(\frac{µ\text{g}}{\text{g}}\right)=\frac{{\text{A}}_{451}\;\text{x}\;\text{Volume}\;\left(\text{ml}\right)\;\text{x}\;10^{4}}{{\text{A}}^{1\%}{}_{1\text{cm}}\;\text{x sample weight}\;\left(\text{g}\right)}$$

where, A451 = absorbance at 451 nm; Volume = total volume of extract (1.25 mL), A^1%^_1cm_ = absorption coefficient of a carotenoid. The mean absorption coefficient for various carotenoids [28] was calculated as 2701. Sample weight = 0.05 g (almost equivalent to 50 μL of homogenized extract).

#### Visual and digital data collection of fruit sections

Freshly collected fruits were longitudinally sectioned, and sections were scanned using HP Scanjet 4890 PhotoScanner at 300 dpi with a black background. All scanned sections were visually assessed for defining shape categories: flat, rectangular, ellipsoid, obovoid, round, oxheart, long and heart [29]. Obtained images were analyzed using Tomato Analyzer software Version 2.1.0.0 (TA) (The parameters examined are listed in S1 Table). Fruit size and shape were analyzed based on few parameters explained by Brewer et al [30] and color parameters as explained by Darrigues et al [31].

### Statistical methods

#### Univariate statistical analysis

Mean, standard deviation (SD) and coefficients of variation (CV) were calculated for 5 selected parameters (fruit weight, fruit shape (VD/HD), brix, total carotenoids, a*/b*) of the fruits from 127 accessions, based on manual as well as tomato analyzer measurements. The accessions with values > (mean + SD) and > (mean + 2SD) were identified as desirable and highly desirable ones, whereas, those with values < (mean–SD) and < (mean– 2SD) were identified as undesirable and highly undesirable ones. All others were considered as with average performance [32].

#### Clustering of accessions

63 parameters (phenotypic subcategories mentioned in S2 Table) were used for clustering 127 accessions using Jaccard’s coefficients as a measure of similarity by agglomerative hierarchical clustering. Clustering was also done using Pearson’s coefficient for 55 fruit quantitative parameters (49 parameters collected by TA as given in S1 Table and 6 manually collected parameters; fruit weight, vertical diameter, horizontal diameter, pH, °Brix and total carotenoids). The cophenetic correlation was used as a measure of goodness of fit for a cluster analysis by NTYSIS software. The degree of fit was interpreted subjectively as follows: 0.9 ≥ r (very good fit); 0.8 > r < 0.9 (good fit); 0.7 > r < 0.8 (poor fit) and r < 0.7 (very poor fit) [33].

#### Factor analysis

The 49 fruit characters used in this study were analyzed in SPSS software v.21 (IBM SPSS version 21.0; IBM Corp., Armonk, NY). Orthogonal rotation of the factor axes (Varimax rotation) was used to extract factors having Eigenvalues > 1. The factors were classified according to the fruit traits.

#### Correlation biplot analysis

A principal component analysis was performed for 26 fruit traits (based on factor analysis) of 127 accessions. The trait-by-accession correlation biplot was constructed. The vectors in the biplot were used to study the correlation between the fruit parameters. Moreover, pairwise Pearsonʹ s correlation coefficients at two significant levels (P<0.05 and P<0.01) were calculated for all combinations of the fruit parameters.

#### Discriminant analysis

Fruit shape parameters collected from TA were taken for stepwise discriminant analysis to classify the tomato accessions on the basis of their shape in SPSS v.21.

### Screening for SNPs

Large-scale DNA extraction was carried out using a protocol described by Sreelakshmi et al [34]. The target genes sequences were obtained from SGN database [10, 11]. The SNP detection was carried out using TILLING protocol described by Blomstedt et al [35] with few modifications. The target gene region was amplified using nested PCR with M13 tailed primers. Primer pairs were designed using the primer 3 software [36, 37] for each selected target sequence (S3 Table). Fluorescent labeled M13 forward (IRDye 700 nm) and reverse (IRDye 800 nm) tails were attached to the 5´ ends of the forward and reverse primers respectively. The first step PCR amplification was carried out with DNA of different accessions mixed with that of Arka Vikas in a 1:1 ratio. The PCR in 20 μL consisted of 5 ng of template, 1X PCR buffer (10 mM Tris, 50 mM KCl, 1.5 mM MgCl2, 0.1% (w/v) gelatin, 0.005% (v/v) Tween-20, 0.005% (v/v) NP-40, pH 8.8), 2.5 mM each dNTPs, 2.0 mM MgCl2, 0.18 μL Taq polymerase (in-house isolated) and 3 pmoles (0.15 μM) each of forward and reverse primers. The cycling conditions for amplification were 94°C-4 min, 35 cycles of 94°C-20 sec, 60°C-45 sec, 72°C-2 min, 72°C-10 min. The first step PCR products were re-amplified using a combination of 0.29 pmoles (0.015 μM) of unlabeled forward primer, 0.42 pmoles (0.02 μM) of IRD700 M13 forward primer, 0.20 pmoles (0.01 μM) of unlabeled reverse primer and 0.50 pmoles (0.025 μM) of IRD800 M13 reverse primer. The cycling conditions for amplification were 94°C-4 min, 5 cycles of 94°C-20 sec, 58°C-45 sec with a decrement of 2°C per cycle 72°C-1 min 30 sec, 30 cycles of 94°C-20 sec, 50°C-45 sec, 72°C-1 min 30 sec, 72°C-10 min followed by heteroduplex formation: 98°C-10 min, 80°C-20 sec, 70 cycles of 80°C-7 sec with a decrement of 0.3°C per cycle and held at 4°C.

The mismatch cleavage reaction was performed in a total volume of 45 μL containing 20 μL PCR product, 1X CEL I digestion buffer (10 mM HEPES buffer pH 7.0, 10 mM KCl, 10 mM MgCl2, 0.002% (v/v) Triton X-100 and 10 μg/mL BSA) and CEL I enzyme at 1: 300 dilution (1 μL/300 μL CEL I digestion buffer). The mixture was incubated at 45°C for 15 min and cleavage reaction was stopped by adding 10 μL stop solution (2.5 M NaCl, 75 mM EDTA, pH 8.0 and 0.5 mg/mL blue dextran). The DNA was precipitated by addition of 125 μL of cold absolute ethanol and a brief incubation at -80°C followed by centrifugation at 4500 rpm in a SH-3000 rotor for 30 min. The DNA pellet was washed with 70% (v/v) ethanol and after drying at 80°C, was suspended in 8 μL formamide loading buffer (37 (v/v) deionized formamide, 1 mM EDTA and 0.02% (w/v) bromophenol blue). The PCR products were denatured by heating at 94°C for 2 min and then were incubated on ice. About 0.5 μL of the sample was electrophoresed in a denaturing 6.5% (w/v) polyacrylamide gel in TBE buffer (89 mM Tris, 89 mM boric acid, 2 mM EDTA, pH 8.3) at 1500 V, 40 mA and 40 V setting on LI-COR 4300 DNA Analyzer. The two TIFF images of 700 and 800 channels were analyzed in Adobe Photoshop software (Adobe Systems Inc.) and were visually assessed for mutations.

### Calculation of SNP frequency

The accessions showing mutation were amplified by the same set of primers as described above with the difference that only unlabeled primers were used for amplification in the second step PCR. The amplicons thus obtained were sequenced using Big Dye Terminator Cycle Sequencing Kit Ver. 3.1 (Applied Biosciences) by Bioserve Biotechnologies (India) Pvt. Ltd. The sequence variations (Substitutions and Indels) were detected by aligning the sequences with wild-type sequence using ‘Multalin’ software [38, 39]. The SNP frequency per Kb was calculated using the formula: (Total number of SNPs detected/ total length of the screened fragment) X 1000 [40]. DnaSP (DNA sequence polymorphism), was used for the analysis of DNA polymorphism from nucleotide sequence data [41, 42]. The nucleotide diversity, Pi (π) and number of haplotypes were calculated by DnaSP. Transition, transversion and Indels proportion was calculated using TASSEL v. 5.1.0 [43, 44]. PARSESNP (Project Aligned Related Sequences and Evaluate SNPs [45, 46] was used to analyze and display the variation in gene sequences and SIFT (sorting intolerant from tolerant) [47] was used to predict the deleterious variations for protein functions. Mega 4 was used to construct the bootstrap consensus (10000 replicates) phylogenetic tree based on sequence polymorphism by neighbor-joining method. Neutrality tests were carried out in DnaSP and Mega4 [48].

## Results

### Analysis of a diverse collection of tomato accessions

A total of 127 accessions from three sources (NBPGR, India; IIVR, India; and TGRC, USA; S4 Table) were analyzed for variability. These accessions were phenotyped at different developmental stages using 16 major and 63 minor morphological categories (S2 Table) corresponding to architectural, vegetative and reproductive variations in tomato with reference to a commercially grown cultivar Arka Vikas (AV) (S1 Fig). Notwithstanding the fact that many accessions were similar to AV, several accessions showed varied phenotypes.

### Maximal diversity was confined to branching/other growth pattern

**Fig 1:**
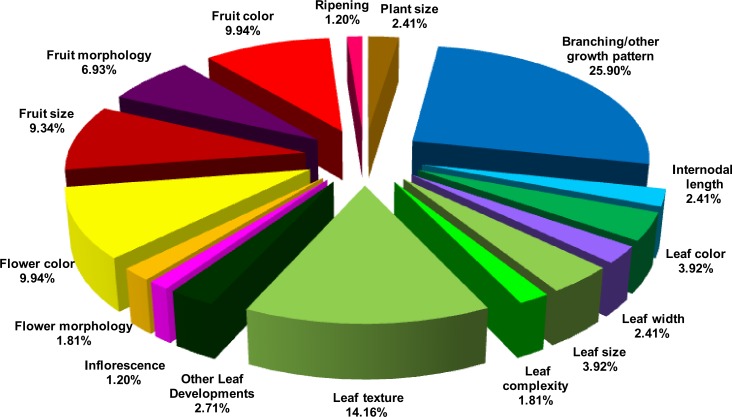
Overall morphological variations observed in tomato accessions. Pie diagram represents percent contribution of each character towards total variability to reference cultivar, Arka Vikas.

Expectedly, maximum variations were in branching/other growth pattern (25.90%) followed by leaf texture (14.16%) (Fig 1). Little variation among the accessions was observed for plant size, only few accessions showed either tall (2.36%) or short (3.94%) plant stature. Likewise, a small number had either long (3 accessions) or short internodes (5 accessions). Consistent with the primary function of leaf to carry out photosynthesis, variation from green color such as yellowish green or pale green leaf (10.24%), or abnormal leaf width (6.3%), or leaf size (10.24%) was seen in few accessions. Interestingly, two accessions had simple leaf and four accessions displayed intermediate leaf, in contrast to normal compound leaf character of tomato. The flowering time in the majority of accessions was nearly similar to AV. The flower color showed more diversity with pale or strong yellow colored flower phenotypes (25.98% accessions). However, only minor variations were discernible (4.72%) in floral morphology with flowers displaying varied characters such as broad petal, or varied floral organ size, or small flowers. Similarly small sized inflorescences were observed in few (3.15%) accessions.

The fruits displayed wide diversity with variations in color, shape and size (S2 Table; S2 Fig). In 24.41% of the accessions, the size of ripe fruit was different from AV. While small fruited accessions mostly belonged to indigenous collections, the big fruited accessions largely belonged to TGRC (14 out of 16). A wide diversity was observed in fruit shape with 18.11% showing round, deformed, fused, striped, notched, oval or pumpkin-shaped fruits. Likewise, ripened fruits (~25.98%) showed distinct color variations like red, dark red, orange, pink, light pigmented and striped pattern (S3 Fig; S2 Table). Remarkably, a minimal diversity was observed in ripening behavior except four accessions (3.15%) with non-ripening phenotype (*nor*, *Nr*, *rin* and EC 398684).

The overall morphological similarity among accessions based on Jaccard’s coefficients ranged from 0.192–0.875. Agglomerative hierarchical clustering by unweighted pair-group method of arithmetic averages (UPGMA) grouped the accessions on the basis of 63 morphological parameters representing overall plant diversity (Listed in S2 Table) into 14 classes (S4A Fig). Among these, the Class1 comprised of 80 accessions including AV, followed by Class 7 with 18 accessions (S4B Fig). Interestingly one accession EC35322 (*Abd*, Hungarian landrace) was an outlier with only ~22% similarity. The cophenetic correlation coefficient (CCC) between similarity matrix and UPGMA phenogram was rMORP = 0.85 for qualitative morphological traits. The relative high CCC value indicated a good fit of the cluster analysis to the dataset.

### Morphometric analysis reveals wide diversity in fruit shape

During domestication, tomato cultivars have been preferentially selected for bigger fruit size. Consistent with this, the fruit weight varied in accessions ranging from 1 g (BL-1208) to 334 g (LA1996) compared to ~58.00 g weight of AV. Most accessions with higher fruit weight than AV belonged to the TGRC collection. (S5 Fig and S6 Fig). Expectedly, the fruit diameter largely correlated with the fruit weight, however, the ratio between vertical (VD) to horizontal diameter (HD) varied indicating variation in the fruit shape. The majority of accessions had round shaped fruits (VD/HD = 0.8–1.20) followed by squat (ratio <0.8) and elongated (ratio >1.20) fruits, respectively (S5 Fig and S6 Fig). Arka Vikas bore squat fruits (ratio = 0.74). Among the eight fruit shape types recognized by [29] (S7 Fig), the oxheart and long shapes were not present in the accessions, and majority of fruits were of flat shape (flat>round>heart>obovoid>rectangular>ellipsoid) Digital analysis of cut halves of fruits brought forth more detailed information regarding fruit shape and color (S8 Fig).

The characteristic red color of tomato fruit signifies completion of ripening. The cut halves of fruits using CIEL*a*b* color system [49] were examined for average red, green, blue, luminosity, L*, a*, b*, hue and chroma (S8J Fig). All the above parameters showed distinctive variations among accessions. One divergent accession was *hp1* mutant (LA3538) with least value for average Luminosity, L* and b*, in contrast to the nonripening *rin* and *nor* mutants that were at the opposite end of the scale. Consistent with the absence of carotenoid accumulation, the a* value was low in *rin* (LA1795) mutant and highest in *B*^*og*^ (LA4025) mutant. In agreement with its nonripening phenotype, the a*/b* ratio was also least in *rin* mutant (LA1795).

The morphometric analysis of the fruits was supplemented with the examination of basic parameters of ripening such as level of total carotenoids, acidity and total soluble solids of fruits. The total carotenoid content varied widely among the accessions with almost 15 fold difference between the lowest (11.71 μg/g, LA1016, *dps (diospyros) L*. *esc*.) and highest (177.72 μg/g, LA4040, *S*. *pennellii* IL 2–5) level (S5 Fig and S6 Fig). In the table and processed varieties of tomato, the acidity of fruits normally ranges between pH 4–5, and the same was observed for the majority of accessions (68.5%). The notable accession was *nor* mutant (LA3770) with pH 2.2 signifying that absence of ripening is likely associated with the high acidic value. Interestingly, homogenates of two accessions were alkaline (EC398704, EC-50-50) with the extreme value of pH 8.6 in EC-50-50 (S5 Fig and S6 Fig). Similar to acidity, the total soluble solids (TSS)/°Brix also showed wide variation ranging from 1.8 (EC27885; a landrace from Ghana) to 7.5 (LA4025; *B*^*og*^) (S5 Fig and S6 Fig).

**Fig 2:**
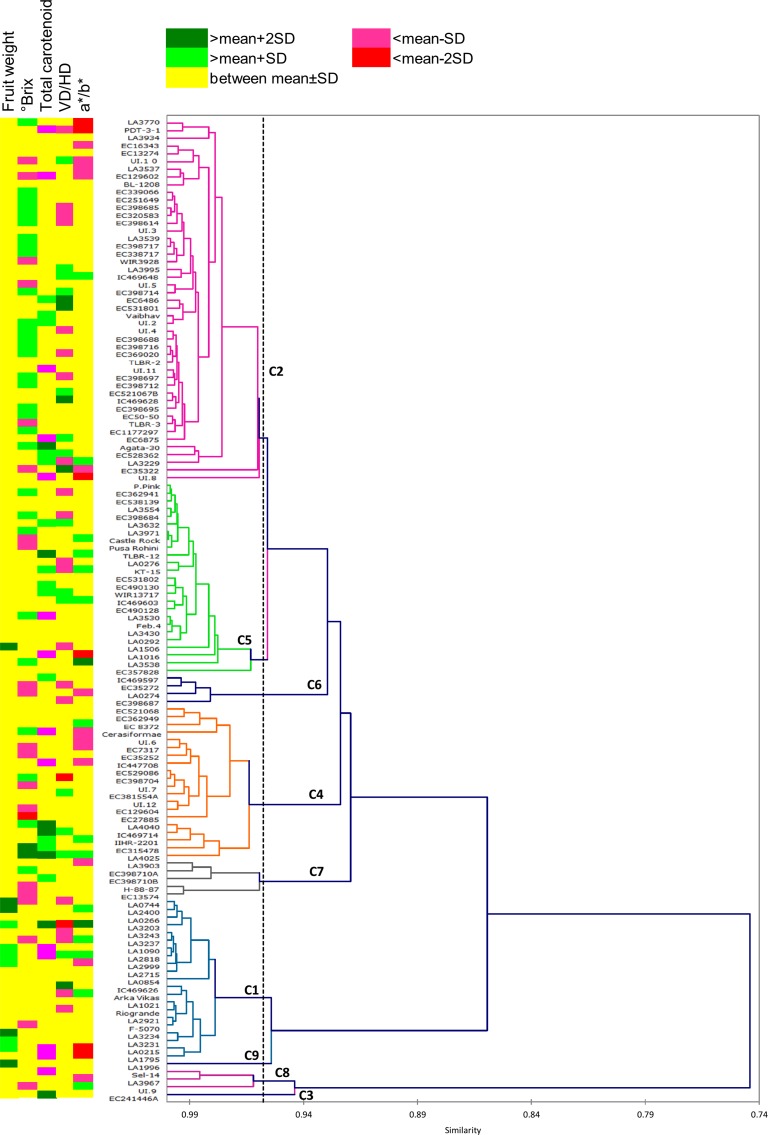
Clustering of the tomato accessions on the basis of 55 fruit attributes. Agglomerative hierarchical clustering by unweighted pair-group average method was used for clustering. The accessions were grouped into 9 classes (C1-C9). Each class is represented by a different color in the dendrogram. The dotted line on dendrogram represents the position of truncation for defining the 9 classes. The heat map on left side of dendrogram shows the relative values of five selected fruit attributes.

Similarity among fruit phenotypes ranged from 0.31–0.99 based on Pearson coefficients. The accessions were clustered into nine classes using 55 fruit attributes (Fig 2). In contrast to vegetative morphological traits, the cophenetic correlation coefficient for fruit traits, rFRUIT = 0.68, was moderate. The different classes formed upon clustering were populated with a variable number of accessions ranging from 47 for class 2 and only one each for class 3 and 9. The variance within the class was 22.38% and between the classes was 77.62%. The class 3 and 8 were outliers signifying their distinct fruit phenotype from others. The class 1 which also included AV was populated with several commercially cultivated accessions indicating a likelihood of close relationship amongst these accessions.

The performance analysis with 6 selected fruit parameters revealed that fruits of class 1 and 9 were squat with highest fruit weight and ripening index indicator (a*/b*) but low TSS and carotenoids (S5 Table). The class 3 was opposite to these two classes with round fruits, lowest fruit weight, and a*/b*, and highest TSS and carotenoids levels. Class 6 comprised of round fruits with average carotenoids level but least pH and TSS, whereas Class 8 fruits had lowest carotenoids level with round fruits.

#### Fruit shape is a complex trait

The 49 fruit parameters (as listed in S1 Table) were further subjected to factor analysis to identify highly correlated variables. A total of 12 factors having an Eigenvalue >1 (based on Kaiser’s criterion) accounted for a cumulative variance of 86.59% (S6 Table). The proportion of each variable’s variance that could be explained by the extracted factors is depicted by communalities (S1 Table). All the variables scored high (except two shape variables: distal angle micro and heart shape had values <0.5) indicating the usefulness of the factors in representing the variables in the analysis.

Factor 1 was strongly associated with variables such as area, perimeter, height and diameter of the fruit and was termed as the trait representing “**size**” (Table 1). All variables associated with factor 1 showed very high positive factor loadings. On the other hand, “**shape**” attributes were associated with nine factors (2,3,5,7,8,9,10,11 and 12). The contribution to “**shape**” was quite complex as several variables were parallelly associated with 2 or more factors. For example, proximal fruit end shape showed positive factor loadings on Factor 2 and 3, whereas distal fruit end shape attributes loaded positively on factors 2 and 11 (Table 1). The rotated component matrix depicted many shape variables had +ve factor scores whereas others had–ve factor scores, thus emphasizing a complex correlation among the morphometric parameters determining fruit shape. The “**color**” trait included all modules of TA that loaded on factors 4, 6 and 10. One of the factors, Factor10, was shared by color (chroma) and shape (distal fruit end shape) variables.

**Table 1:** Fruit traits associated with the factors extracted from factor analysis.

S.N.	Fruit traits	Factors	Correlation between the factor and variables	
			+ve	-ve
A	Size	1	Basic measurement	
B	Shape	2	Proximal fruit end shape	Fruit shape index
			Distal fruit end shape	
		3	Proximal fruit end shape	Homogeneity
			Latitudinal section	
			Blockiness	
		5	Asymmetry	Asymmetry
		7	Internal eccentricity	Internal eccentricity
		8	Blockiness	Blockiness
		9	Latitudinal section	
		10	Distal fruit end shape	
		11	Homogeneity	
			Asymmetry	
		12	Distal fruit end shape	
C	Color	4	a*	Hue
			a*/b*	Green
				b*
		6	Green	
			Red	
			Blue	
			L*	
			b*	
			Luminosity	
		10	Chroma	

* is part of the Lab color space represented by a, b, and L

#### Fruit shape- whether round or flat?

The factor analysis revealed shape to be a complex trait. Fruit shape variables were subjected to stepwise discriminant analysis to classify the accessions as per the categories defined by Rodríguez [29] (S7 Fig). Taking cognizance that sample size of the smallest group should exceed the number of variables for this analysis, only two groups- round (41) and flat (69) were included in this analysis. The assumption of equality of covariance matrices was validated by the log of group's covariance matrix and within-group covariance, which was equal to each other. Out of 37 variables (1–37 variables listed in S1 Table), only 6 were used for discriminating the two groups- fruit shape index external.1, height mid-width, circular, lobedness degree, horizontal asymmetry, ovoid and proximal indentation area (S7A Table). Only 1 discriminant function (DF1) with an eigenvalue of 3.247 and canonical correlation of 0.874 was sufficient in explaining 100% variance between round and flat shapes, and Wilks’ lambda indicated a highly significant function (p < .000) (S7B and S7C Table).

The standardized canonical discriminant coefficients of each of the 6 predictor variables are enlisted in S7D Table. Height mid-width, circular and lobedness degree positively correlated to DF1 whereas fruit shape index external.1, proximal indentation area and horizontal asymmetry ovoid were negatively correlated. The classification was cross-validated by “Leave- One- Out Classification” that correctly classified 99.1% accessions (S7E Table).

#### Correlation among fruit traits

A trait-by-accession biplot for 26 selected parameters on 127 accessions is depicted in S9 Fig. Strong correlations were observed for color attributes hue, chroma and b*; and total carotenoid and a* (representing color); fruit weight and area (representing basic measurements) and pericarp area and thickness (representing latitudinal section). Proximal indentation area (representing proximal fruit end shape) and ellipsoid (representing the TA category, homogeneity) were negatively correlated. Fruit shape index was negatively correlated to fruit shape triangle and distal fruit end blockiness but positively correlated to pericarp area and thickness. Negatively correlated variables were asymmetry- ovoid and obovoid; homogeneity- rectangular and ellipsoid and color- a* and b*. Pearson’s correlation coefficients show expected correlation such as a positive correlation between fruit weight and area (0.763); pericarp area and thickness (0.710) and a negative correlation between L* and a* (-0.587) (S8 Table).

#### Leads available- desirable accessions for crop improvement programs

Parameters influencing marketability of tomato fruits such as weight, shape, color and quality (TSS) were specifically considered for identifying superior germplasm accessions. Classification of desirable and undesirable accessions based on a combination of the mean and SD for each attribute as outlined by Shakhatreh et al [32] was followed (S9 Table, Fig 2). The redness of fruit as visualized by a*/b* showed 6 accessions with highly undesirable fruits and these turned out to be the non-ripening mutants of tomato. The accessions such as *hp1*, AV, *sp*, *B*^*og*^, had high a*/b* value. Total carotenoid ranged from 11.71 μg/g FW (LA1016; *dps*. L.esc.) to 177.72 μg/g FW (LA4040, *S*. *pennellii* IL 2–5) with 7 accessions being highly desirable. Only two accessions (1.57%) qualified as highly desirable for TSS, whereas, 30 (23.62%) were in the desirable range.

In addition, consumers may have differential preferences for two of the parameters, fruit weight, and shape, depending on their individual needs. The collection of fruits in our study varied from large to small fruited types with squat, round and elongated shapes. Considering large fruited individuals to be desirable, we identified 6 accessions as desirable (4.72%) and 5 as highly desirable (3.93%) for fruit weight (S9 Table) whereas rest showed average fruit weight. Arka Vikas had average fruit weight of ~58 g in comparison to others. In contrast to this, breeding programs which require developing small-sized fruit varieties can consider small fruited types in this collection as desirable. Similarly, for fruit shape (VD/HD) which was in the range of 0.58–1.48, either of the three types (squat, round and elongated) can be considered as desirable as per the downstream breeding purposes. The fruits with value 0.79–1.16 were almost round whereas <0.79 (22 accessions) were squat and >1.16 (19 accessions) were elongated in shape.

### Genetic variability in selected genes regulating fruit development is low

Single nucleotide polymorphisms (SNPs) were analyzed in a set of 10 genes in various plant developmental processes in tomato by using mismatch cleavage of heteroduplexed DNA on Li-COR DNA Analyzer (S10 Table, S10 Fig). Among these five genes [*1-aminocyclopropane-1carboxylate synthase 2* (*ACS2*;Solyc01g095080.2.1), *Lycopene beta cyclase* (*CYC-B*; Solyc06g074240.1.1), *ripening inhibitor* (*RIN*;Solyc05g056620.1.1), *non-ripening* (*NAC-NOR*;Solyc10g006880.2.1), and *phytoene synthase1* (*PSY1*; Solyc03g031860.2.1)] play a role in fruit development and ripening. The other four genes [*CONSTITUTIVE PHOTOMORPHOGENIC 1 homolog* (*COP1*; Solyc12g005950.1.1), *Phototropin1* (*PHOT1*; Solyc11g072710.1.1), *phytochrome A* (*PHYA*; Solyc10g044670.1.1), *phytochrome B1* (*PHYB1*; Solyc01g059870.2.1)] are involved in light perception and signaling. Recent evidences have indicated that light perception in tomato clade has an influence on leaf shape [50] and time measurement during fruit ripening [51]. The *MSH2* gene was used as a control as it does not have a direct role in plant development. Above screen identified accessions with SNPs/indels in all genes except, *PSY1* and *RIN*. (Table 2, S11 Fig). A total of 43 polymorphic sites and 11 Indels were identified leading to 36 nonsynonymous changes in the protein coding region whereas the rest 18 were silent in nature. Maximum nonsynonymous changes were seen in *COP1* whereas none was observed in *PHYB1* illustrating the highly conserved nature of *PHYB1* gene. Conversely, *PHYB1* accumulated a maximum number of synonymous changes followed by *PHYA*. In all other genes, synonymous changes were not observed. The proportion of total changes accounted by nucleotide transitions, transversions, and Indels was estimated to be 0.423, 0.365 and 0.211 respectively (S11 Table). The maximum transitions were accounted by G→A followed by A→G whereas maximum transversions were of type A→T and G→T. Among Indels, deletions of two types (A:—and C: -) were found in *NAC-NOR* gene whereas insertions of three types (-: A, -: C and -: G) were seen in *COP1* gene.

**Table 2:** Nucleotide polymorphism.

Gene	Chromosome	bpScreened	Coding Region (%)	Individuals carrying SNPs	Haplotypes	Nucleotide Diversity (π)	Nature of SNP		Changes			Zygosity	SNP/Kb
									Protein coding region		Non coding region		
							Polymorphic sites	Indels	Non-synonymous	Synonymous			
***ACS2***	1	1687	59	3	4	4x10^-5^	11	0	3	0	8	Hetro	0.051
***COP1***	4	3520	63.02	22	6(4)*	8x10^-5^ (0.303)**	9	6	11	0	4	6 Homo /9 Hetro	0.034
***CYC-B***	12	1231	82.23	2	3	3x10^-5^	2	0	2	0	0	Homo	0.013
***MSH2***	6	1243	37.92	1	2	4.48x10^-6^	2	0	1	1	0	Homo	0.013
***NAC-NOR***	10	942	53.09	7	4(3)*	3x10^-5^(0.091)**	2	5	7	0	0	Homo	0.059
***PHOT1***	11	898	20.05	1	2	2x10^-5^	11	0	10	0	1	Homo	0.096
***PHYA***	10	2802	69.18	2	3	1x10^-5^	3	0	2	1	0	Hetro	0.008
***PHYB1***	1	868	25.18	1	2	4.68x10^-6^	3	0	0	3	0	Hetro	0.027
***PSY1***	3	1119	26.95	0	1	NA	0	0	NA	NA	0	NA	NA
***RIN***	5	1947	33.08	0	1	NA	0	0	NA	NA	0	NA	NA

*: Indel haplotypes

**: Indel diversity[k(i)]

**Fig 3:**
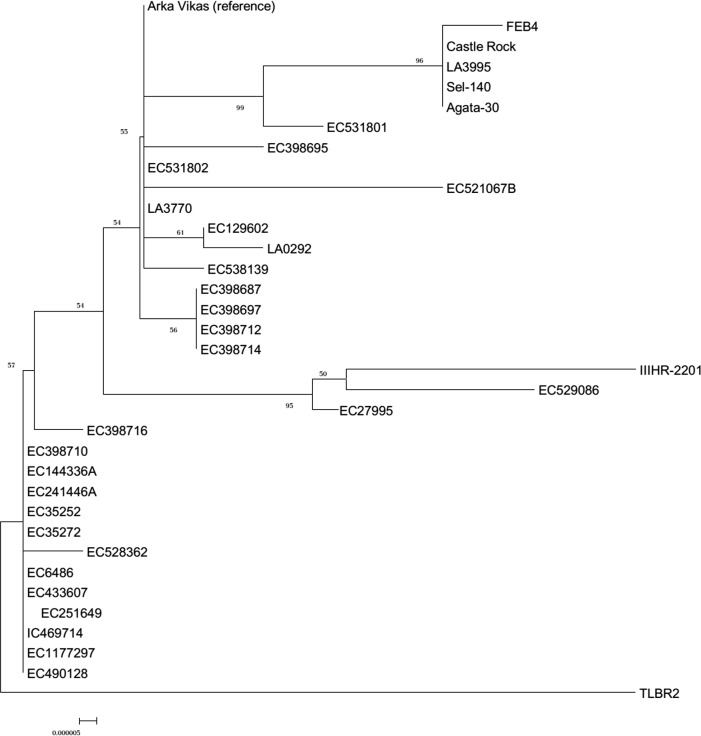
Clustering of accessions based on SNP polymorphism. The tree was made using the Neighbor-Joining method in MEGA4 taking together the sequences of all the genes. Substitutions are represented by branch lengths and the bootstrap value is indicated as numbers above the branches (10000 replicates).

The overall nucleotide diversity, π, was low and varied from 4.48 x 10–6 in *MSH2* to 8 x 10–5 in *COP1*. Indels identified in two of the genes, *COP1* (6) and *NAC-NOR* (5) lead to an Indel diversity, k(i), of 0.303 in *COP1* and 0.091 in *NAC-NOR*. Bioinformatic analyses of the sequences revealed the existence of these polymorphisms either in heterozygous or in homozygous states. *COP1* polymorphisms exhibited both kinds of zygosity. The frequency of SNP/Kb varied from gene to gene and was minimum in *PHYA* (0.008) and maximum in *PHOT1* (0.096). The average frequency of polymorphism across the genes is 0.038/Kb. Dendrogram by Neighbor-Joining method (Fig 3) with reference to Arka Vikas showed that TLBR-2 and IIHR-2201 had the maximum polymorphism in the population.

#### EcoTILLING identified haplotypes in genes screened

A total of 28 haplotypes were identified from amplicons of 10 selected genes with their number ranging from 1–6 (S12 Fig). The haplotype with similarity to AV (reference haplotype) was the largest group. The frequency of reference haplotype varied from 0.83 (*COP1*) to 1.0 (*PSY1* and *RIN*). One or more NBPGR accessions grouped as non-reference haplotype(s) in all genes except *PHOT1*, *PSY1*, and *RIN*. The overall frequency of non-reference haplotypes was rare occurring at levels <0.05 except *COP1* and *NAC-NOR* (0.17 and 0.055, respectively). The minor allele frequencies in analyzed genes were rare (<0.05) except for two SNPs in *COP1* which were in complete linkage disequilibrium (data not shown).

#### Evolutionary insights into nucleotide diversity

DNA sequences which evolve randomly (neutral) can be differentiated from nonrandomly evolving ones by Tajima’s D neutrality tests. A value of 0 denotes neutral evolution whereas a negative or positive value denotes a deviation from the neutral theory of evolution. All the genes tested for the neutrality showed a negative value (S12 Table). The Tajima’s D test statistic in two genes, *ACS2*, and *PHOT1* was significant and highly significant, respectively, implying the presence of rare alleles in low frequency or recent selective sweep or a recent bottleneck followed by population expansion. Indeed, the minor alleles in *ACS2* and *PHOT1* were present in just 4 accessions in the population (3 for *ACS2* and 1 for *PHOT1*). The rest of the genes showed non-significant values. Contrary to this, Tajima’s D (Nonsyn/Syn) for the coding region in *PHYA*, was 1.344 but was nonsignificant in a test of neutrality by Mega 4.

Another indicator of selective pressures on protein coding sequences can be ascertained by the Ka/Ks ratio which is the number of nonsynonymous substitutions per nonsynonymous site (Ka) to the number of synonymous substitutions per synonymous site (Ks). The test result for *PHYA* revealed Ka/Ks value <1 indicative of purifying selection.

## Discussion

Post-domestication, the cultivation of tomato has been adopted across several environmental zones and cropping systems with more than 10,000 cultivars being grown worldwide. The above adoption was facilitated by breeding tomato cultivars adapted to growth constraints, disease and pest resistances and fruit productivity and quality [52] and was assisted by introgression from wild relatives [53] [54] resulting in wide phenotypic diversity. In recent years the analysis of genomes of several of tomato cultivars/heirlooms has provided insight into the genetic diversity of tomato; nonetheless the linkage of genetic diversity with phenotypic diversity remains to be achieved. One major challenge is to interpret how the genetic variations among the cultivars are translated to the functional and phenotypic diversity. Compared to information on genomic diversity, little information is available for the quantitative characteristics of phenotypes [55]. Comparative phenotyping of a large number of diverse cultivars accompanied with the usage of statistical correlation tools to link with the genome(s) can provide linkage between genotype and phenotype [56]. Such an analysis using primary metabolome of tomato introgression lines harboring *S*. *pennellii* genome revealed several QTLs that regulate the carbohydrate content in tomato fruits [57].

Examination of 127 tomato accessions revealed wide diversity in both vegetative and reproductive characters. On the basis of morphological traits, the accessions clustered into 14 classes with a high CCC value indicative of minimal distortion in observed similarity matrices. The vegetative plant habit showed the maximal variance followed by leaf texture and flower color while floral morphology varied only a little. Though the pedigree of most tomato accessions is not known, it is likely that many of these variations in plant habit and leaf texture may in part arise from introgression of genes from wild relatives [58]. The analysis of genome-wide SNP data of tomato cultivar Heinz 1706 revealed introgressions from *S*. *pimpinellifolium* genome into chromosomes 4, 9, 11 and 12 of tomato [7].

The molecular networks regulating phenotype diversity in vegetative habits are not yet known. An extensive study on tomato leaf traits reported 1035 QTLs associated with it and also revealed that leaf shape and size has a negative relationship [59] [60]. A comparative study on the evolution of leaf shape in tomato with reference to its wild progenitors revealed that a complex gene regulatory network regulates leaf morphology in tomato [61]. Thus, the wide diversity of SNPs in regulatory genes present in tomato cultivars may likely contribute to above morphological variations [61] [9]. The observed little variation in floral morphology in tomato cultivars is consistent with the reduced diversity in nucleotide polymorphism found in genes regulating floral meristem compared to rest of tomato genome [62].

Similar to leaf morphology, the fruit characters showed a wide diversity in color, weight, and shape. Morphological diversity in cultivated tomato fruit ranges from small to large, round, blocky, elongated, pear-shaped fruits, with color ranging from red to green, white, black, pink, orange or yellow whereas wild tomatoes are known to bear small, round red or green fruits. A study using 331 red-fruited tomato accessions involving fruit weight and other traits classified above accessions into three groups viz. PIM, CER, and BIG. The genomic analysis of accessions revealed that PIM as the ancestor of BIG with CER as the evolutionary intermediate [9]. In the current study, the accessions were clustered into nine fruit classes, however, in contrast to vegetative traits; the CCC for fruit traits was moderate. Interestingly, the class one was mainly populated by commercially grown tomato cultivars, indicating the close relationship among the members. The fact that class one members also had maximal fruit weight and ripening index indicator indicate that these accessions are likely to be similar to BIG class. On the other hand, the class three with low fruit weight and high carotenoid level likely represent the PIM group.

Among the fruit traits, the shape appeared to be more complexly regulated as the largest number of factors was associated with it. Moreover, several of these factors were also cross associated with other fruit parameters. Above observation is consistent with complex genetic regulation of fruit morphology in tomato where at least seven loci (*fw2*.*2*, *fw3*.*2*, *lc*, *fas*, *ovate*, *sun* [63] and *elf1* [64] have been reported to regulate the fruit shape and weight [65]. Among these loci, *lc* and *fas* regulate locule number and flat fruit shape, whereas *sun* and *ovate* regulate elongated fruit shape [29]. During domestication of tomato, two different sets of loci contributed to increase in fruit mass, wherein, first set of five loci contributed to the domestication and second set of thirteen loci contributed to improvement leading to two-step evolution of tomato fruit mass [9]. Among the fruit shape variation, the maximal accessions belonged to round and flat fruit category. Post-anthesis the variation in fruit growth pattern contribute to the shape variation, fruits with uniform growth are round whereas asymmetry in growth leads to a flat fruit [63]. The step wise discriminant analysis of fruit shape using six parameters showed a high canonical correlation of discriminant factor 1 with round and flat shape. Considering that large numbers of loci have contributed to the evolution of fruit mass in tomato [9] the asymmetry/symmetrical fruit growth may have a similar genic contribution. The fruit color also showed a complex regulation with multiple factors as several loci including light signaling regulate the formation of carotenoids in tomato fruits [66] [51].

The fruit trait TSS, an important character for selection of processing varieties of tomato, was associated with two factors. It is reported that phenotype difference between processing varieties of tomatoes and other tomatoes is related to genomic variations located on chromosome 5 in most cultivars [9]. Among the fruit traits, TSS appears to be a trait that is least influenced by other traits. Similarly, Panthee et al [67] also reported that TSS correlated only with flavor and acidity.

In tomato, fruit weight and the number of fruits per inflorescence are key traits selected by the breeders in the tomato parental lines to produce high yielding hybrids [68]. The positive and negative correlations between different fruit traits highlighted the complexity of interaction between the fruit traits. The traits such as TSS, carotenoids, and fruit mass and shape influence each other in positive as well in negative fashion highlighting that breeding for one trait may comprise other trait. The genetic analyses have indicated that most of these traits are quantitative in nature, thus are likely influenced by multiple genes interacting with each other in myriad fashion [69] [65].

In tomato, much of observed phenotypic diversity has come from the selection of varieties that suits an agro-climatic zone coupled with suitability for fresh market or processing industry [70]. Compared to phenotype diversity, the diversity in genotypes is much lower. The variations observed in fruit color and carotenoids were wide which did not correlate with the polymorphism observed at the molecular level. The analysis of nucleotide diversity in a set of nine genes that influences fruit development/ripening and carotenoid levels by either acting as master regulators of ripening (*RIN*, *NOR*), or ethylene biosynthesis (*ACS2*), or light signalling (*PHYA*, *PHYB*, *COP1*, *PHOT1*) or directly contributing to carotenoid biosynthesis (*PSY1*, *CYC-B*) [66] revealed variable degree of polymorphism. One reason for low SNP frequency could be that we mostly examined the SNPs in the exons of above genes, whereas in most tomato accessions, significantly higher SNP frequency is observed in intergenic regions than in genic regions as revealed by genome sequencing [8]. Our results are also consistent with polymorphism in thirteen genes contributing to fruit diversification and plant growth (*ovate*, *fw2*.*2*, *ls*, *og/beta*, *lcy1*, *lfy*, *rin*, *sp*, *fer*, *style*, *psy*, *lin5* and *locus lc (gb|JF284941*)), where low SNP diversity was found [8]. Among above 13 genes, four genes *ovate*, *fw2*.*2*, *lc*, and *fas* specifically regulate fruit shape. The low genetic diversity in these four genes signifies that notwithstanding low genetic diversity a complex intergenic interaction appears to regulate the fruit shape.

The genic polymorphism was either absent or was very little in genes that are essential for fruit development. Moreover, for these genes, the polymorphism was restricted to either intronic regions or it was synonymous in nature. Consistent with this, no SNPs were detected in genes such as *RIN* and *PSY1* that are essentially contributing to the induction of ripening and the first step of carotenoid biosynthesis respectively. The only exceptions were ripening mutants, *nor*, *Nr* and *rin* (LA3770, LA3537 and LA1795) (that have mutations in genes leading to altered/ delayed ripening) and accession, EC 398684. The similar low degree of polymorphism among the old world tomato cultivars was also observed in other studies using either a tomato SNP array [71] or SSR markers [72]. The low degree of nucleotide polymorphism in above genes that are purported to be essential for fruit development is in consonance with similar reduced SNP diversity observed in floral meristem genes [62] which are essential for flower/fruit development in tomato. They also found a strong purifying selection in several of the candidate genes involved in flower and fruit development. In our study, we observed large and negative Tajima’s D for two of the genes, *ACS2*, and *PHOT1*, which indicated rare polymorphisms occurring in low frequency. A probable explanation is that it may be due to the effect of background selection [73], genetic hitchhiking [74] or population size extension following a bottleneck. Nonetheless, the overall nucleotide diversity revealed 26 haplotypes in the genes screened. It would be interesting to unravel the diversity in loci responsible for fruit shape that was inferred as a more complex trait than color.

From the foregoing, it is apparent that linkage between genome and phenome needs a more rigorous analysis. Unlike genome that is mostly fixed for an organism except epigenetic changes, the phenome of the plant involves a more complex expression of genome modulated by developmental homeostasis and agro-climatic influence on above modulation. The phenotyping of plants combined with genome-wide association studies of desirable traits with iterative networking may help to reveal linkages [75]. Xu et al. attempted to link fruit traits with the QTLs using genome-wide association and identified several associations [76]; however, the density of SNP was too low to identify SNPs in candidate genes. In contrast, genome-wide association analysis of metabolic traits in two different studies identified loci associated with few traits, validating known genes as well as deciphering new candidate genes [22] [77].

In summary, our study indicates that while there is low polymorphic diversity in the genes examined by EcoTILLING, the population shows wider phenotype diversity. Since the morphological diversity likely arises from a subtle interaction between genome, transcriptome, proteome and metabolome, the observed phenotype diversity is a manifestation of above interactions. This is also manifested by the fact that rather than single genes, a large number of QTLs [59, 60] and loci [9] regulate leaf shape and fruit size/mass in tomato respectively. Our study indicates that the diversity in fruit traits including pigmentation in tomato cultivars is not similarly manifested in the genes contributing to this response. A more rigorous approach involving a combination of omics with robust bioinformatics tools may decipher the desired genome to phenome linkage assisting plant breeding for desired traits. Taking cognizance of the importance of such an approach, recently DivSeek initiative has been launched to ensure that in the gene banks the genotype and phenotype information are stored along with the seeds for potential use for omics-integrated breeding [78].

## Supporting Information

S1 Fig Morphology of tomato cultivar Arka Vikas used as the reference. Representative images show the whole plant (**a**), compound leaf (**b**), inflorescence (**c**), individual flower (**d**), side view (**e**), longitudinal section (**f**) and transverse section (**g**) of red ripe fruit.
(TIF)

S2 Fig Frequency of accessions showing variability in morphological parameters. Frequencies were calculated from the data collected using PDA, based on visual observations for 15 parameters. The variability in each parameter in three different germplasm sources: NBPGR, IIVR, and TGRC is shown. In each sub-category the reference variety (Arka Vikas) is indicated with an asterisk symbol.
(PDF)

S3 Fig Representative diversity in fruit morphology manifested by different tomato accessions. The fruit phenotypes of Arka Vikas (**a**), LA3530 (**b**), EC 363863 (**c**), LA1016 (d), *S*. *lycopersicum var*. *cerasiforme* (**e**), BL-1208 (**f**), LA0276 (**g**), Agata-30 (**h**), LA1795 (**i**), LA2818 (**j**), Vaibhav (**k**), LA3203 (**l**) are shown.
(TIF)

S4 Fig Clustering of the tomato accessions based on categorical qualitative data. The field-grown plants were phenotyped for 63 morphological parameters. The data was collected using a PDA. Agglomerative hierarchical clustering based on unweighted pair-group method of arithmetic averages (UPGMA) was used for grouping. The accessions were clustered into 14 classes (C1-C14) by automatic truncation (**a**) A 3-dimentional graphical representation of the distribution of accession obtained from different germplasm sources (NBPGR, IIVR and TGRC) in the 14 classes. The classes are indicated on X-axis (1–14) and the number of accessions are plotted in Y-axis. Number of accessions belonging to different germplasm sources are represented with colored bars (Z-axis) and are numerically indicated (**b**).
(PDF)

S5 Fig The distribution of tomato accessions with respect to a range of different fruit traits. **a**-Fruit weight (g), **b**-pH, **c**-VD/HD, **d**-Brix, e-total carotenoids (μg/g FW).
(PDF)

S6 Fig Morphometric and biochemical analyses of different fruit traits in tomato accessions. The distribution of various fruit traits are grouped on the basis of source of tomato accessions. The reference variety Arka Vikas (black) is compared with tomato accessions obtained from NBPGR (red), IIVR (green) and TGRC (blue).
(PDF)

S7 Fig Longitudinal sections of the fruits showing various shape categories of tomato based on the classification by Rodríguez et al. (2011). Two shape categories- oxheart and long were not found in the population used in this study. 41 accessions were classified as round, 69 as flat, 7 as heart, 2 as ellipsoid, 3 as rectangular and 5 as obovoid shaped. The fruit of reference cultivar Arka Vikas was categorized as flat shaped.
(TIF)

S8 Fig Scattergrams showing distribution of various fruit morphology parameters measured using tomato analyzer software. The basic measurements of fruit size (**a**), fruit shape (**b-i**) and fruit color (**j**) are shown. The variables with superscript P and S are plotted with reference to the primary axis (left side y-axis) and secondary axis (right side y-axis) respectively.
(PDF)

S9 Fig Correlation biplot generated by PCA (in XLSTAT) showing the projection of 26 fruit measurement parameters of 127 tomato accessions in the factor space. The variables were selected based on factor analysis. The vectors indicate the parameters. Angle between two vectors explains the correlation between them and the length of each vector shows the variability of the parameter among the accessions.
(PDF)

S10 Fig **Schematic representation of SNP detection in tomato accessions using Eco-TILLING** (**a**); LI-COR image shows the detection of SNPs in *Le-ACS2* gene in tomato accessions (**b**).
(PDF)

S11 Fig Distribution of SNPs detected by EcoTILLING in target genes. Red boxes and lines represent the coding and noncoding sequences respectively. Green boxes represent protein homology block alignments automatically generated using the SIFT program. The PARSESNP analysis shows location of respective SNPs on genomic and coding sequences marked by black, purple and red triangles indicating nonsynonymous, synonymous and nonsense nucleotide substitutions respectively. The positions of Indels are depicted by red squares. The detail of the nucleotide changes, their probable effects and the accession is given along with the PARSESNP output diagram for each gene. PSSM: Position Specific Scoring Matrix (>10:deleterious), SIFT: Sorting Intolerant From Tolerant (<0.05:deleterious).
(PDF)

S12 Fig Haplotype distribution in the selected genes. (PDF)

S1 Table Communalities in Factor analysis: Proportion of variance explained by the extracted factors for each of the variables. Numbers 1–37 and 38–49 represent the shape and color variables digitally collected by TA.
(DOCX)

S2 Table Categories of phenotypic characters and their percentage in the population. (DOCX)

S3 Table List of primers used for screening of SNPs. (DOCX)

S4 Table List of accessions used in the study. (DOCX)

S5 Table Performance analysis of 9 classes for six fruit parameters based on class centroids. The classes were obtained from agglomerative hierarchical clustering (by UPGMA method) of 127 accessions based on 55 fruit attributes.
(DOCX)

S6 Table Total variance as explained by extracted factors in factor analysis. (DOCX)

S7 Table Variables Entered/Removed in the stepwise discriminant analysis of tomato shape (A). Summary of canonical discriminant functions- Eigenvalues (B). Test of significance (C). Standardized Canonical Discriminant Function Coefficients (D). Classification Results of Discriminant Analysis (E).
(DOCX)

S8 Table Pearson’s Correlation between fruit parameters. (DOCX)

S9 Table Univariate statistical analysis for selected fruit parameters of 127 accessions. (DOCX)

S10 Table Annotation of the genes selected for Eco TILLING. (DOCX)

S11 Table Nature of nucleotide changes and its proportion. (DOCX)

S12 Table Neutrality tests. (DOCX)
