# Supplementary figures and images for: Tomato Fruits Show Wide Phenomic Diversity but Fruit Developmental Genes Show Low Genomic Diversity
Source: PLoS One. 2016 Apr 14;11(4):e0152907. doi: 10.1371/journal.pone.0152907 (PMC4831840; doi:10.1371/journal.pone.0152907)

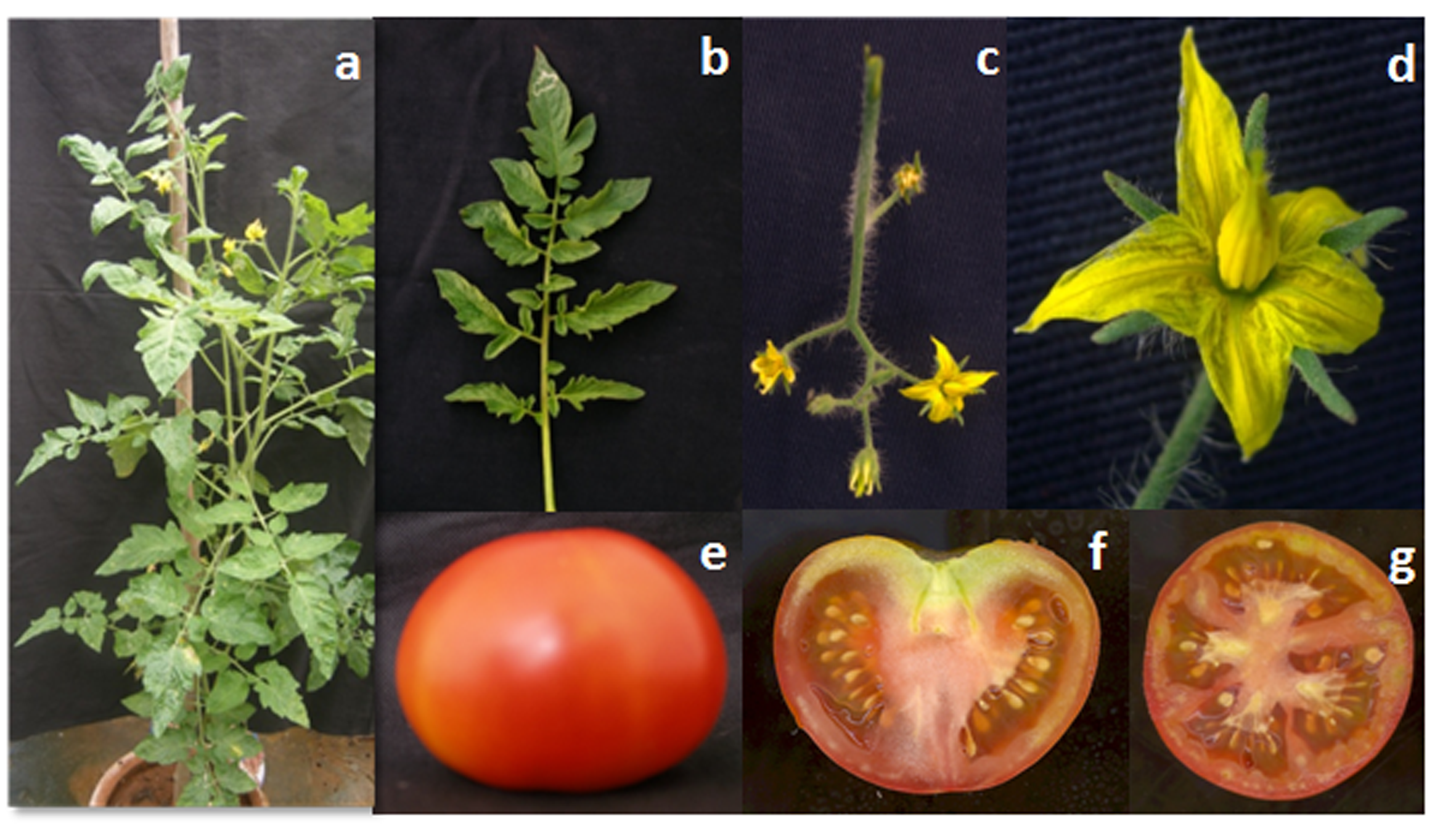

Supplement: S1 Fig — Representative images show the whole plant (a), compound leaf (b), inflorescence (c), individual flower (d), side view (e), longitudinal section (f) and transverse section (g) of red ripe fruit. (TIF) [file pone.0152907.s001.tif]

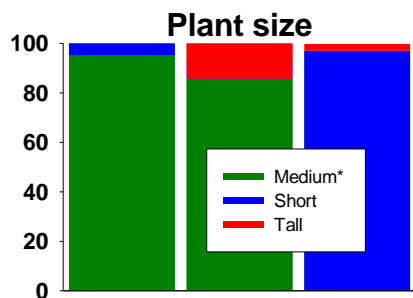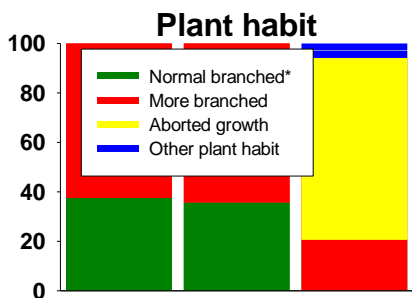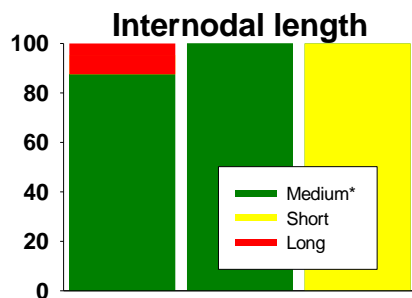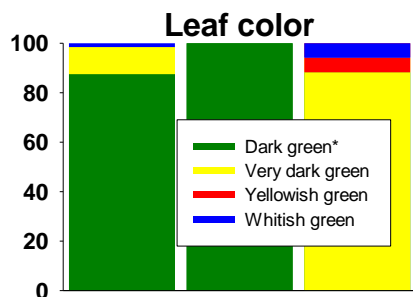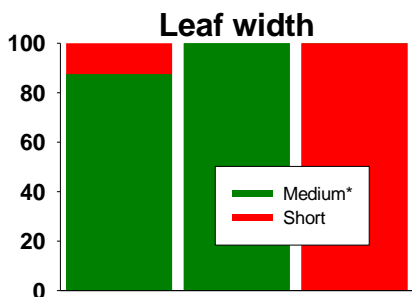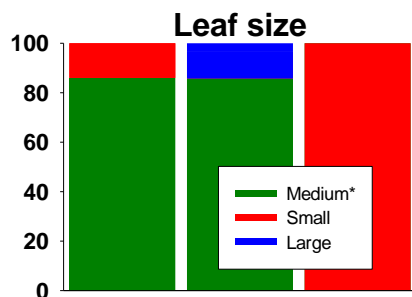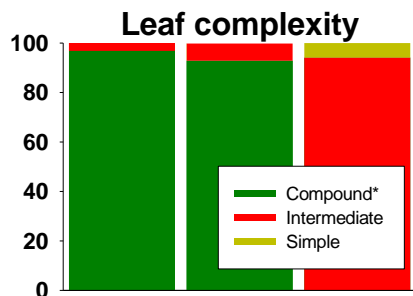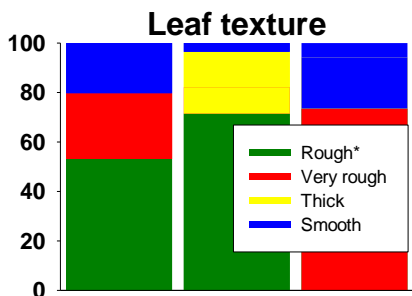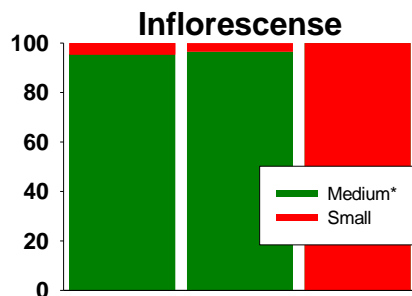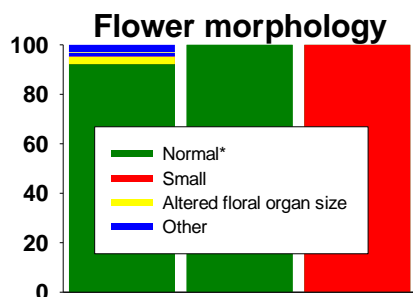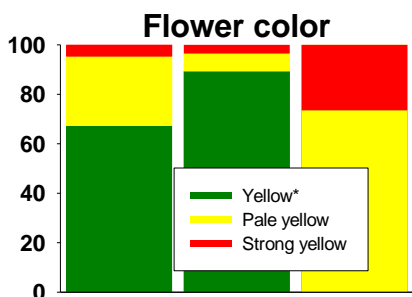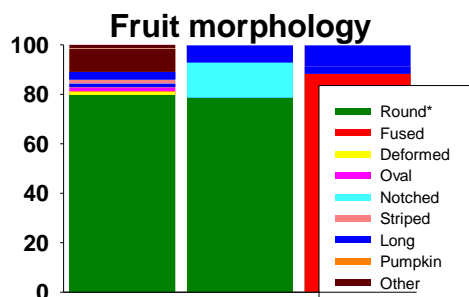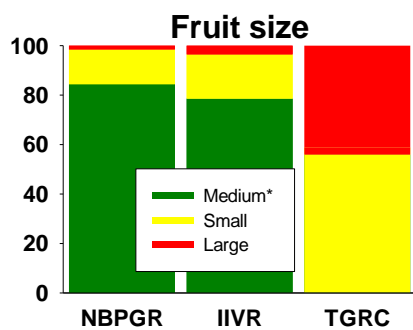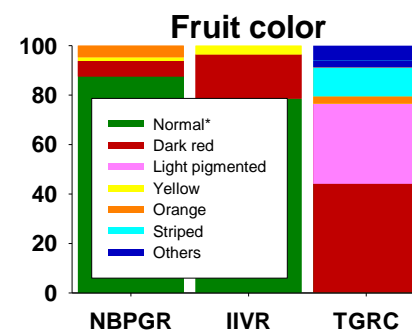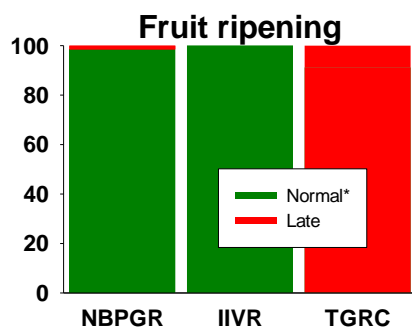

Supplement: S2 Fig — Frequencies were calculated from the data collected using PDA, based on visual observations for 15 parameters. The variability in each parameter in three different germplasm sources: NBPGR, IIVR, and TGRC is shown. In each sub-category the reference variety (Arka Vikas) is indicated with an asterisk symbol. (PDF) [file pone.0152907.s002.pdf]

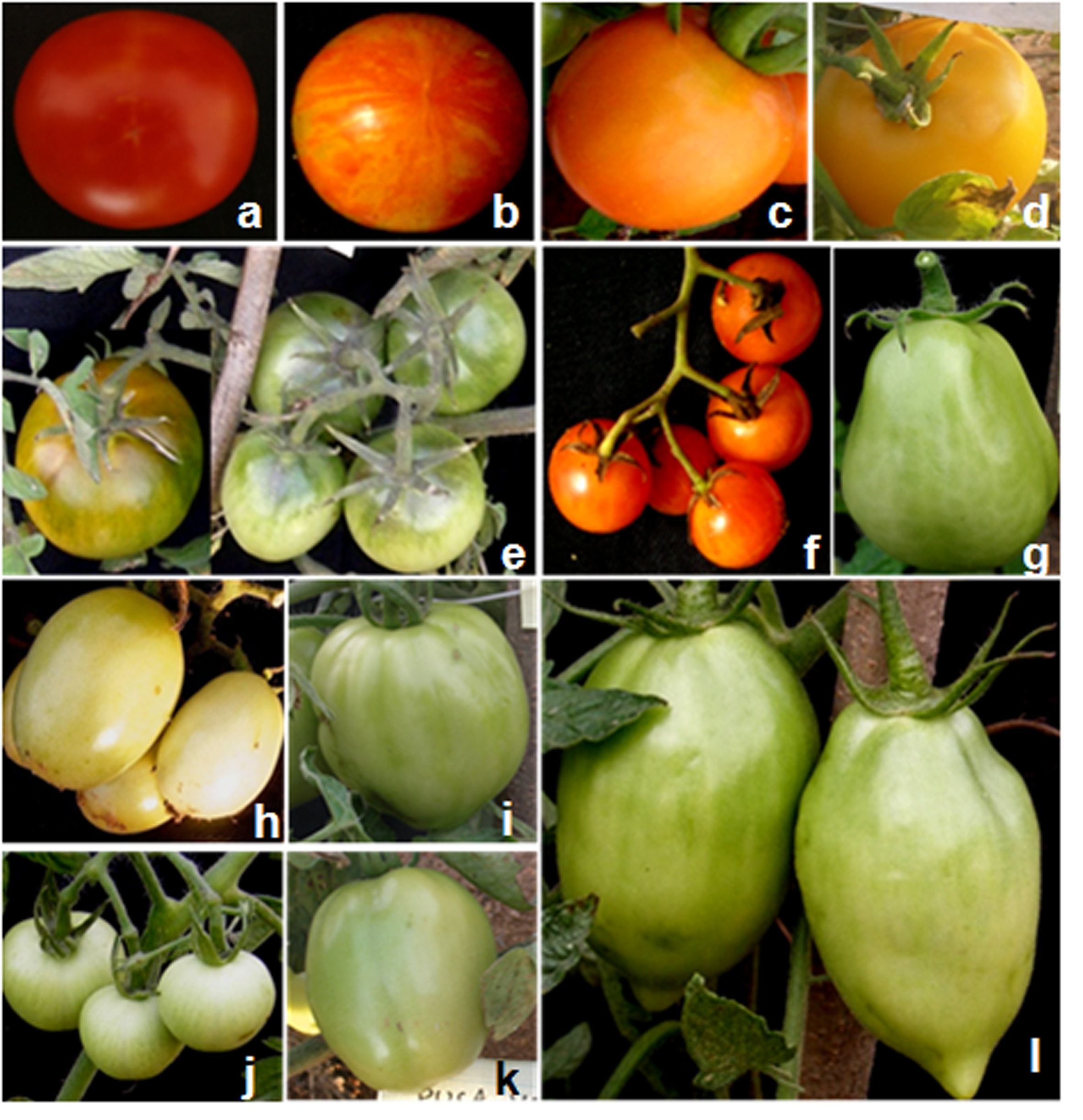

Supplement: S3 Fig — The fruit phenotypes of Arka Vikas (a), LA3530 (b), EC 363863 (c), LA1016 (d), S. lycopersicum var. cerasiforme (e), BL-1208 (f), LA0276 (g), Agata-30 (h), LA1795 (i), LA2818 (j), Vaibhav (k), LA3203 (l) are shown. (TIF) [file pone.0152907.s003.tif]

a

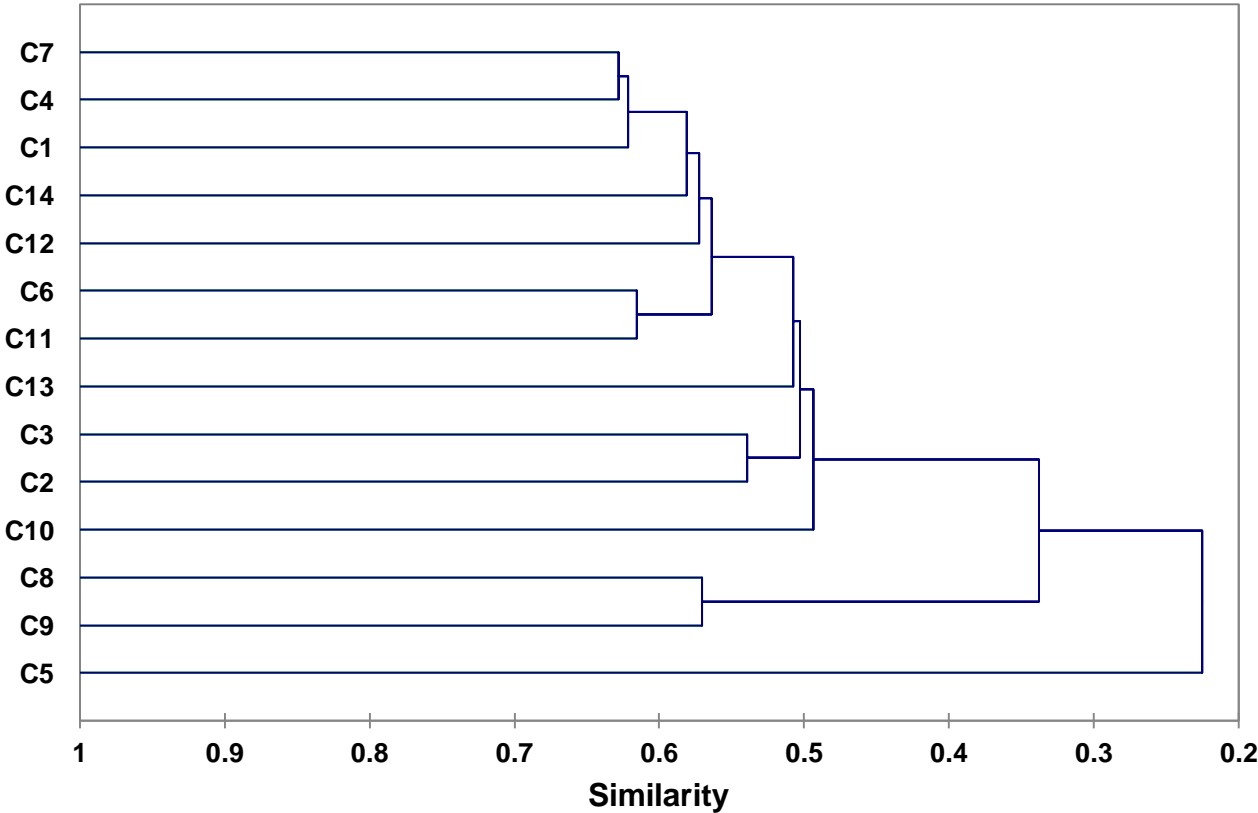

b

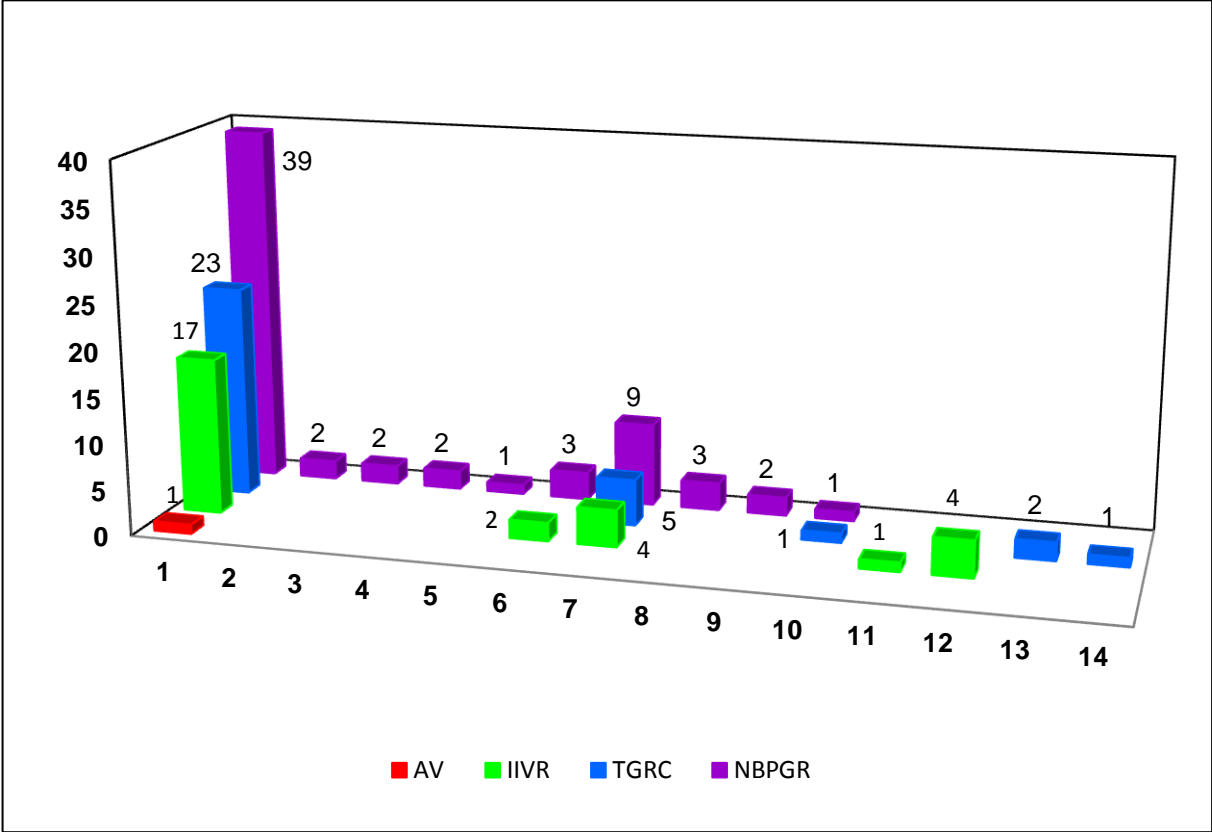

Supplement: S4 Fig — The field-grown plants were phenotyped for 63 morphological parameters. The data was collected using a PDA. Agglomerative hierarchical clustering based on unweighted pair-group method of arithmetic averages (UPGMA) was used for grouping. The accessions were clustered into 14 classes (C1-C14) by automatic truncation (a) A 3-dimentional graphical representation of the distribution of accession obtained from different germplasm sources (NBPGR, IIVR and TGRC) in the 14 classes. The classes are indicated on X-axis (1–14) and the number of accessions are plotted in Y-axis. Number of accessions belonging to different germplasm sources are represented with colored bars (Z-axis) and are numerically indicated (b). (PDF) [file pone.0152907.s004.pdf]

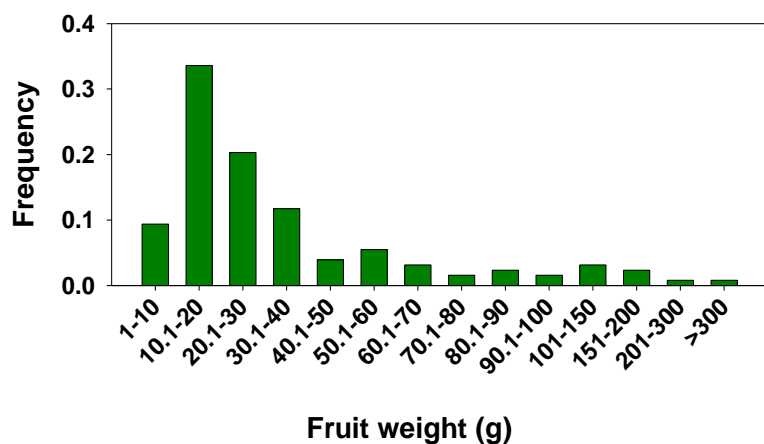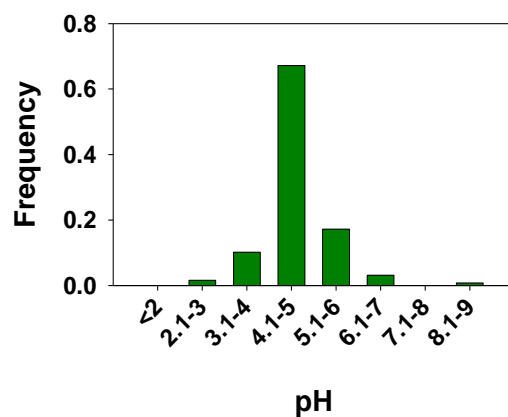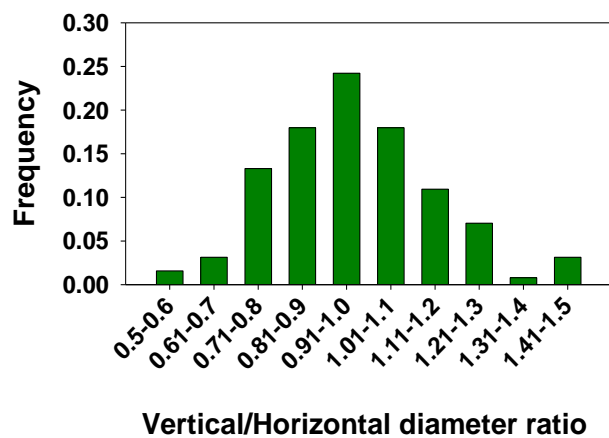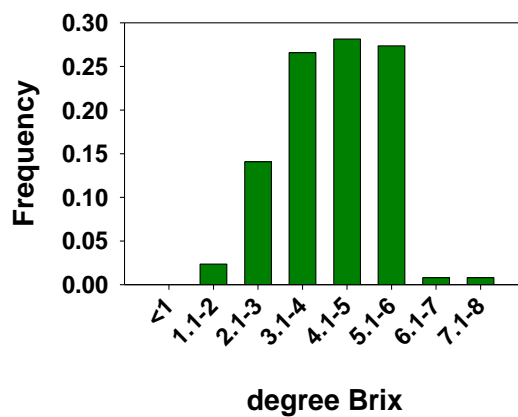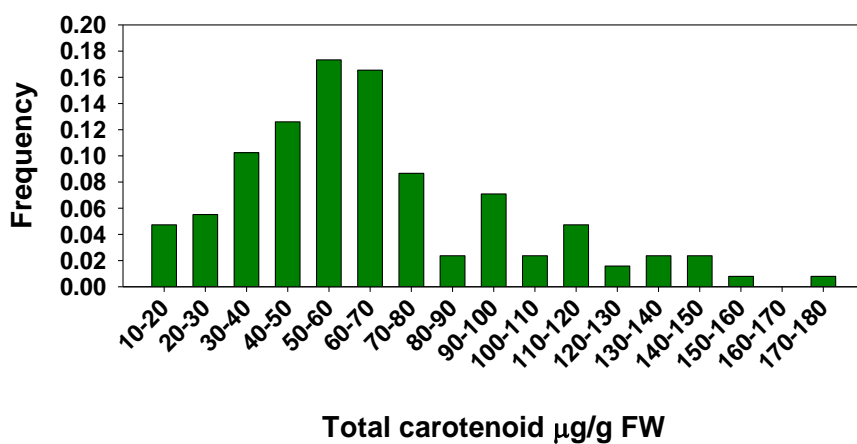

Supplement: S5 Fig — a-Fruit weight (g), b-pH, c-VD/HD, d-Brix, e-total carotenoids (μg/g FW). (PDF) [file pone.0152907.s005.pdf]

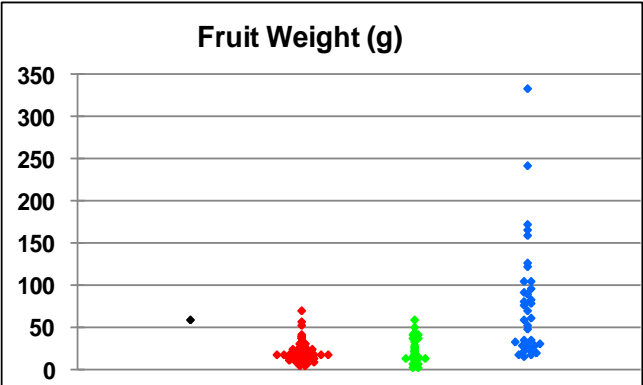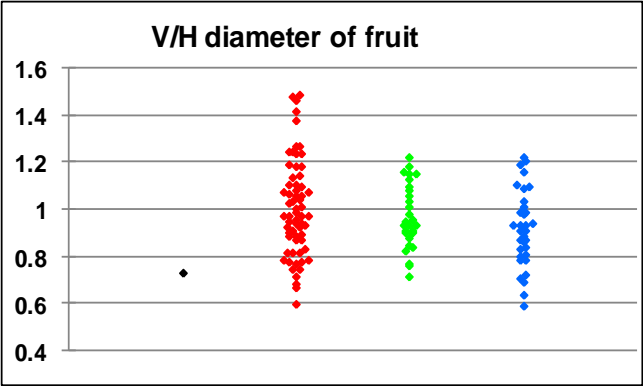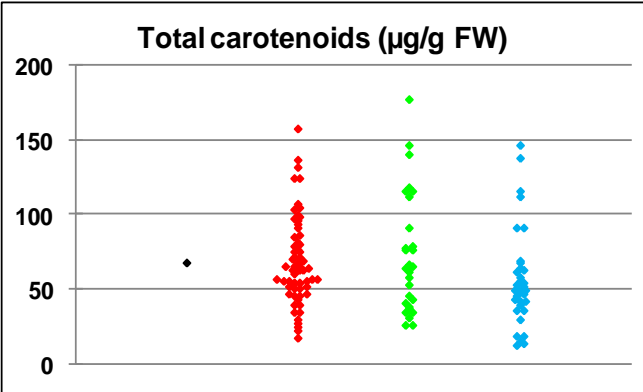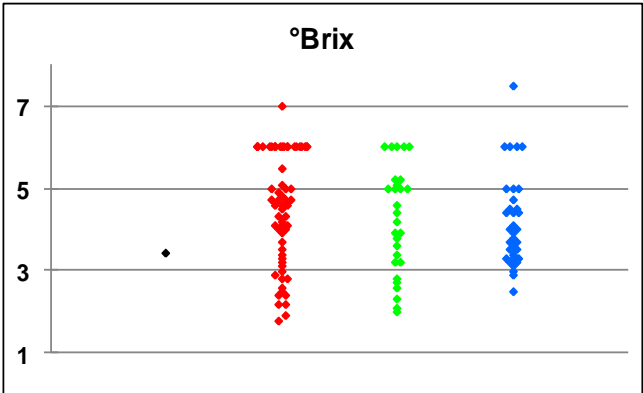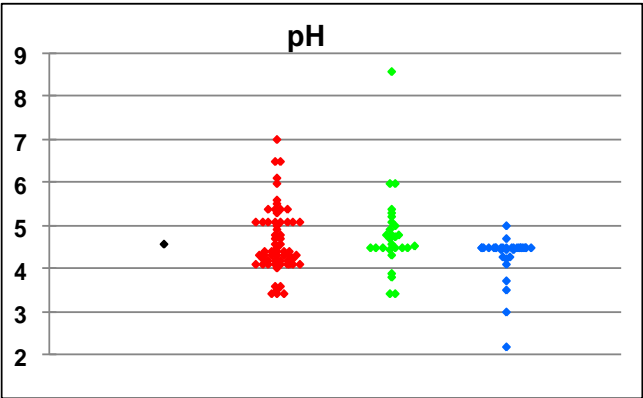

Supplement: S6 Fig — The distribution of various fruit traits are grouped on the basis of source of tomato accessions. The reference variety Arka Vikas (black) is compared with tomato accessions obtained from NBPGR (red), IIVR (green) and TGRC (blue). (PDF) [file pone.0152907.s006.pdf]

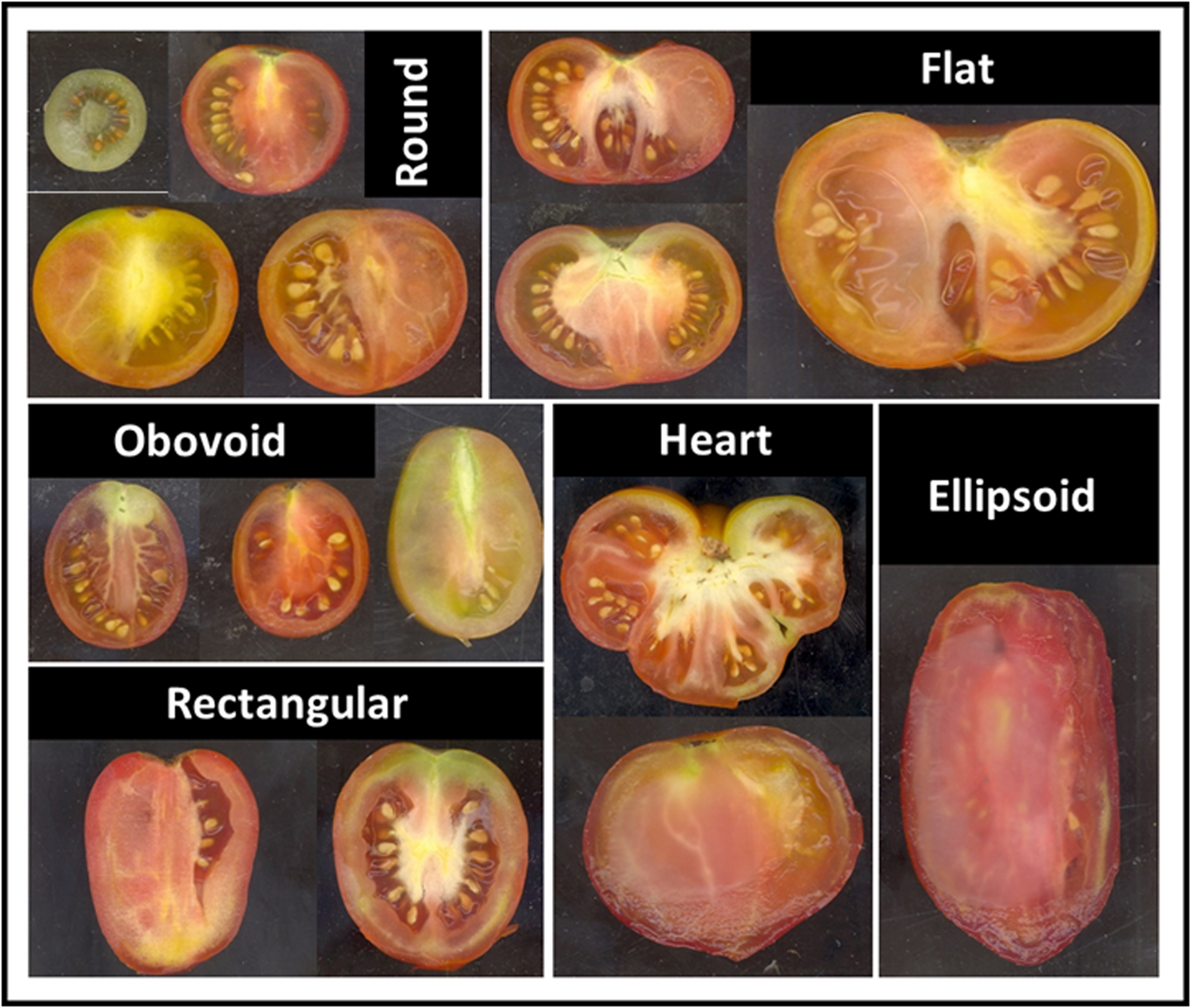

Supplement: S7 Fig — Two shape categories- oxheart and long were not found in the population used in this study. 41 accessions were classified as round, 69 as flat, 7 as heart, 2 as ellipsoid, 3 as rectangular and 5 as obovoid shaped. The fruit of reference cultivar Arka Vikas was categorized as flat shaped. (TIF) [file pone.0152907.s007.tif]

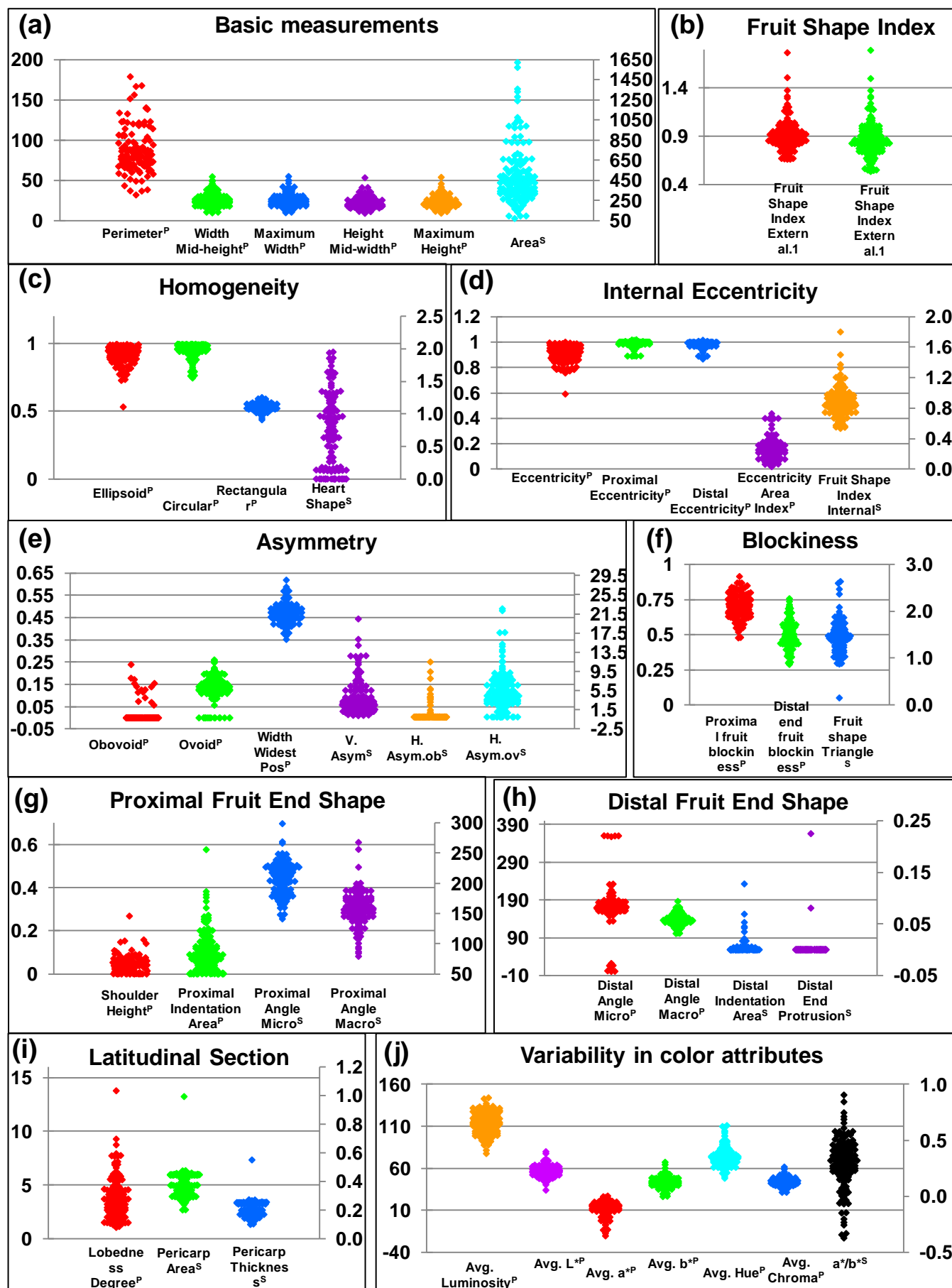

Supplement: S8 Fig — The basic measurements of fruit size (a), fruit shape (b-i) and fruit color (j) are shown. The variables with superscript P and S are plotted with reference to the primary axis (left side y-axis) and secondary axis (right side y-axis) respectively. (PDF) [file pone.0152907.s008.pdf]

Variables (axes D1 and D2: 32.92 %) after Varimax rotation

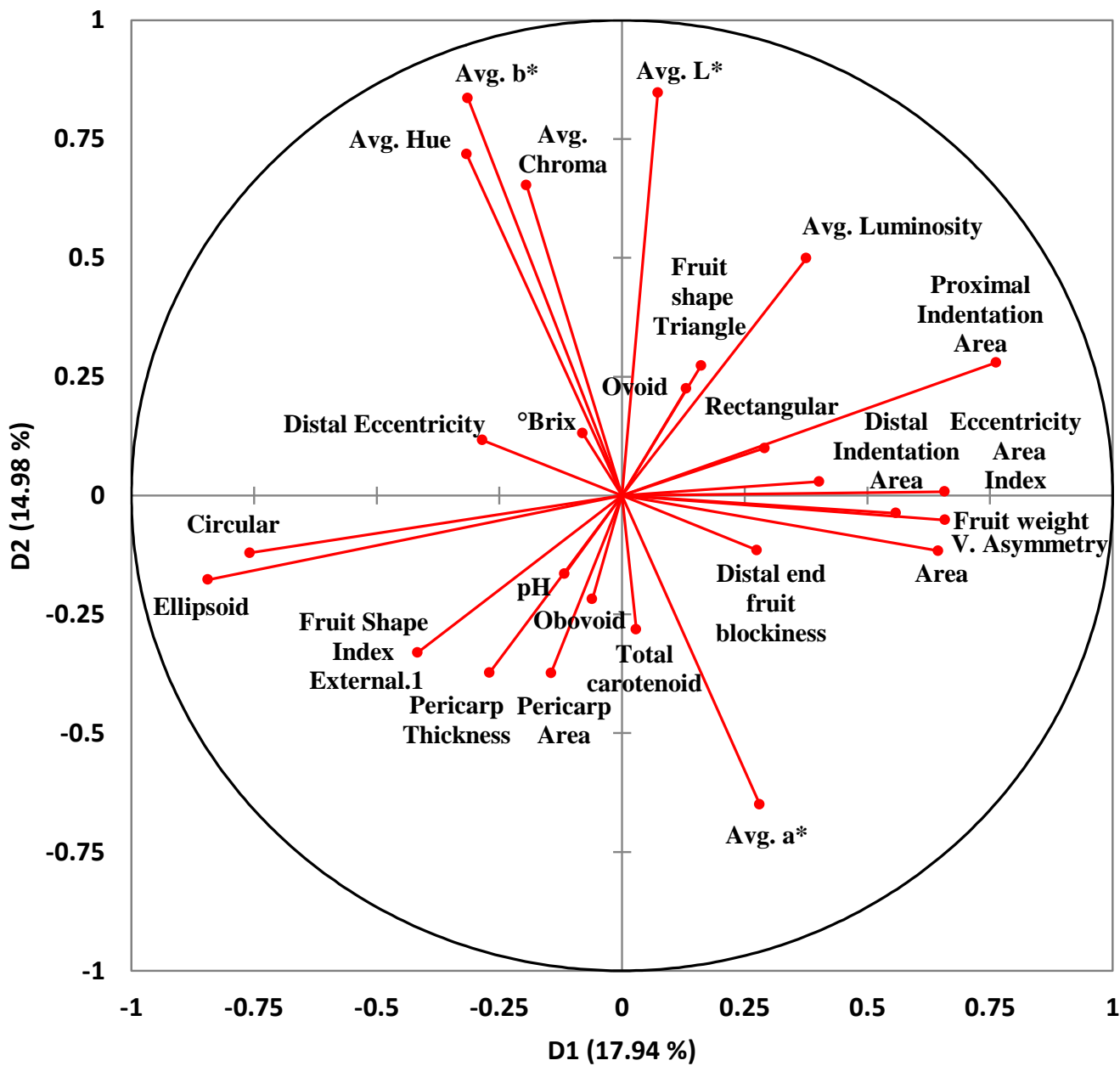

Supplement: S9 Fig — The variables were selected based on factor analysis. The vectors indicate the parameters. Angle between two vectors explains the correlation between them and the length of each vector shows the variability of the parameter among the accessions. (PDF) [file pone.0152907.s009.pdf]

**a**

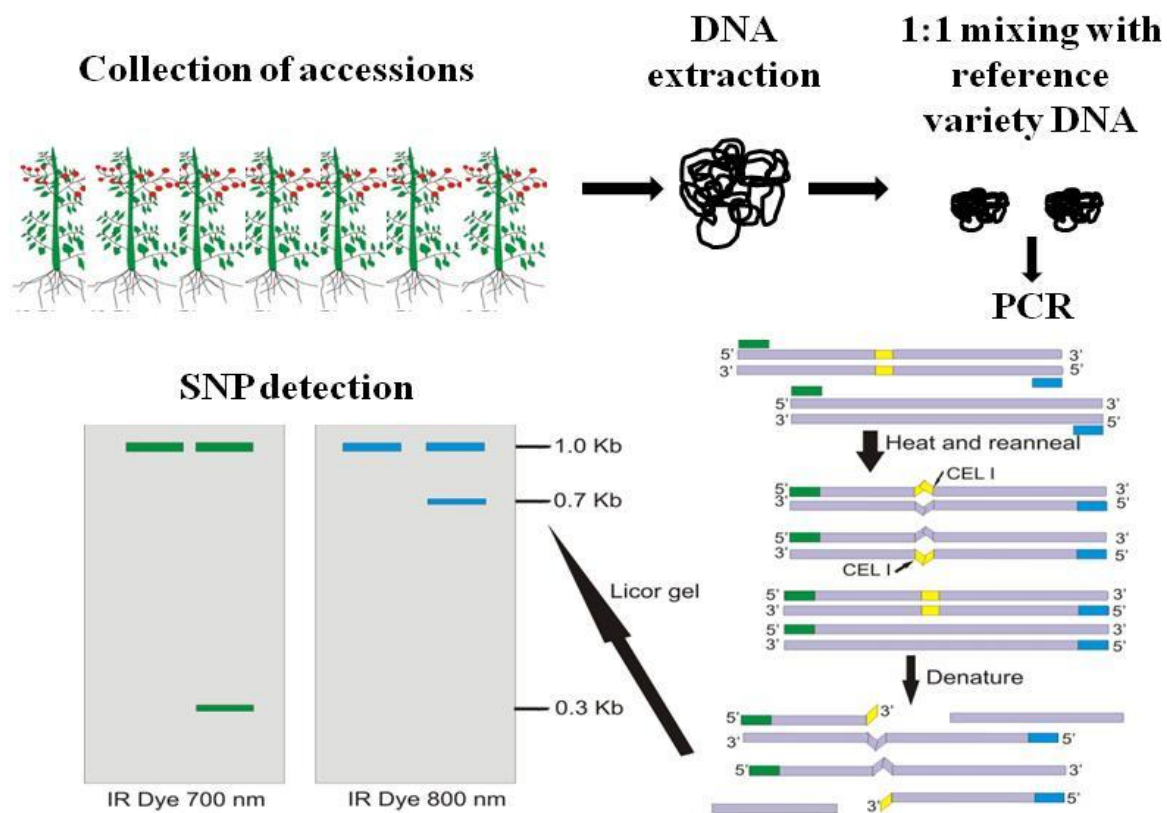

**b**

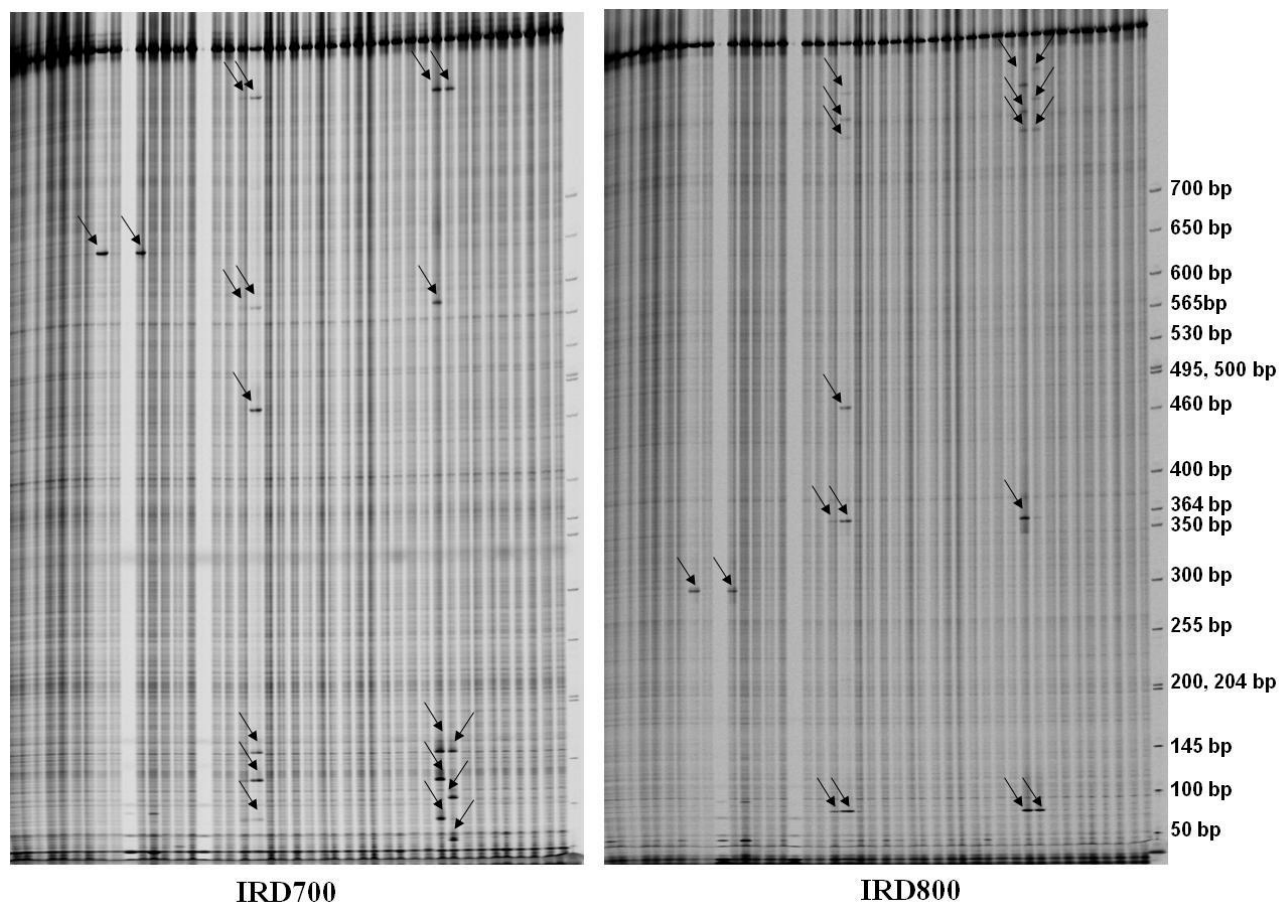

Supplement: S10 Fig — Schematic representation of SNP detection in tomato accessions using Eco-TILLING (a); LI-COR image shows the detection of SNPs in Le-ACS2 gene in tomato accessions (b). (PDF) [file pone.0152907.s010.pdf]

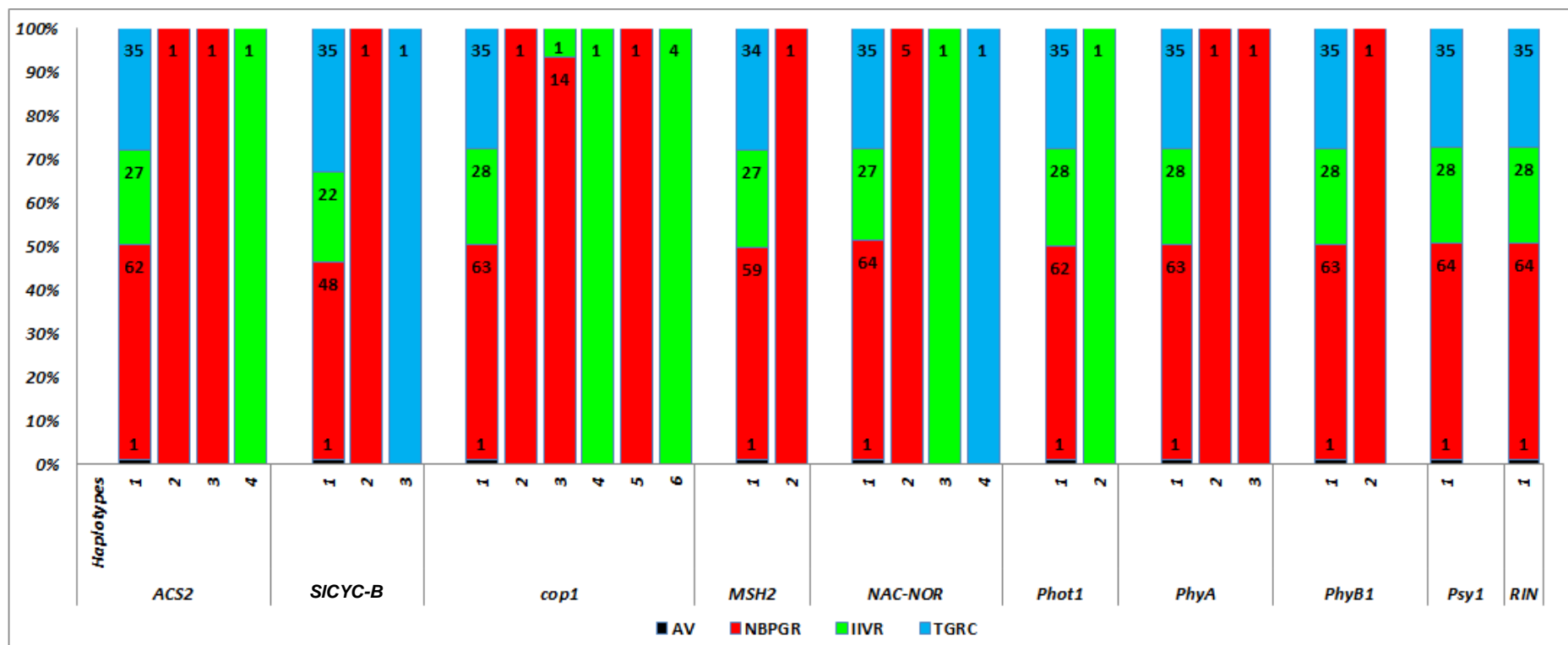

Supplement: S12 Fig — (PDF) [file pone.0152907.s012.pdf]
