# Supplementary material for: Tomato Fruits Show Wide Phenomic Diversity but Fruit Developmental Genes Show Low Genomic Diversity
Source: PLoS One. 2016 Apr 14;11(4):e0152907. doi: 10.1371/journal.pone.0152907 (PMC4831840; doi:10.1371/journal.pone.0152907)
Supplement: S11 Fig — Red boxes and lines represent the coding and noncoding sequences respectively. Green boxes represent protein homology block alignments automatically generated using the SIFT program. The PARSESNP analysis shows location of respective SNPs on genomic and coding sequences marked by black, purple and red triangles indicating nonsynonymous, synonymous and nonsense nucleotide substitutions respectively. The positions of Indels are depicted by red squares. The detail of the nucleotide changes, their probable effects and the accession is given along with the PARSESNP output diagram for each gene. PSSM: Position Specific Scoring Matrix (>10:deleterious), SIFT: Sorting Intolerant From Tolerant (<0.05:deleterious). (PDF) [file pone.0152907.s011.pdf]

ACS2

Genomic Sequence

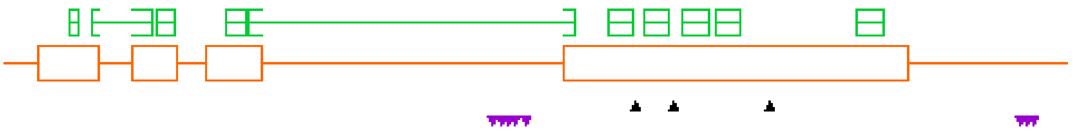

Coding Sequence

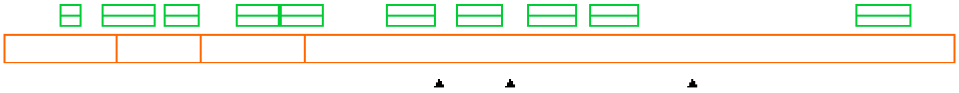

	View On Sequence	Nucleotide Change	Effect	Restriction Enyzme Differences from REBASE		PSSM Difference	SIFT Score	Accession	Zygosity
				Gained in Variant	Lost from Reference				
1	G	A4371G	Intron	MaeIII	TspEI			EC529086	Homo
2	G	C4396T	Intron		MaeIII			IIHR-2201	Homo
3	G	C4396T	Intron		MaeIII			EC529086	Homo
4	G	C4396T	Intron		MaeIII			EC27995	Homo
5	G	G4418A	Intron	HinfI, TfiI	BinI, DpnI, MboI			EC529086	Homo
6	G	A4437G	Intron	Hpy188I				IIHR-2201	Homo
7	G	A4437G	Intron	Hpy188I				EC529086	Homo
8	G	A4437G	Intron	Hpy188I				EC27995	Homo
9	G	G4465A	Intron					IIHR-2201	Homo
10	G	G4465A	Intron					EC27995	Homo
11	G	G4465A	Intron					EC529086	Homo
12	G C	G4786C	V223L	BsmAI		4.0	0.89	IIHR-2201	Homo
13	G C	A4895C	E259A	CviRI, SfaNI	TspDTI	0.8	0.61	IIHR-2201	Homo
14	G C	A4895C	E259A	CviRI, SfaNI	TspDTI	0.8	0.61	EC529086	Homo
15	G C	T5174A	V352E	MboII		-4.5	1.00	EC27995	Homo
16	G C	T5174A	V352E	MboII		-4.5	1.00	IIHR-2201	Homo
17	G C	T5174A	V352E	MboII		-4.5	1.00	EC529086	Homo
18	G	C5898T	Non-coding					IIHR-2201	Homo
19	G	G5919A	Non-coding		DpnI, MboI			IIHR-2201	Homo
20	G	T5937A	Non-coding		AhaIII, MseI			IIHR-2201	Homo

COP1

Genomic Sequence

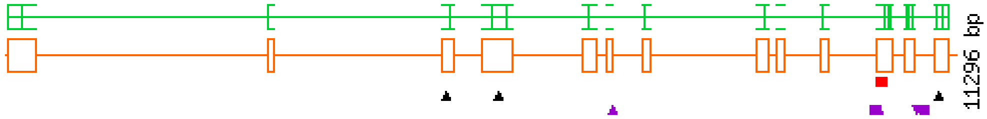

Coding Sequence

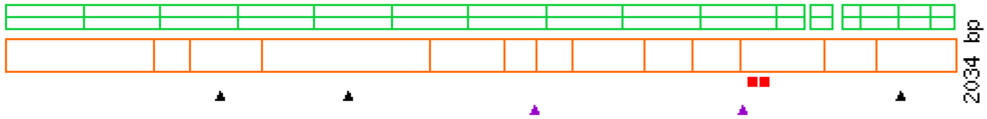

#	View On Sequence	Nucleotide Change	Effect	Restriction Enyzme Differences from REBASE		PSSM Difference	SIFT Score	Accession	Zygosity
				Gained in Variant	Lost from Reference				
1	G C	A5242C	K153T		TspEI	-2.5	0.00	EC398716	Homo
2	G C	A5242C	K153T		TspEI	-2.5	0.00	EC529086	Homo
3	G C	A5242C	K153T		TspEI	-2.5	0.00	TLBR-2	Homo
4	G C	A5242C	K153T		TspEI	-2.5	0.00	EC398710	Homo
5	G C	A5242C	K153T		TspEI	-2.5	0.00	EC490128	Homo
6	G C	A5242C	K153T		TspEI	-2.5	0.00	EC35252	Homo
7	G C	A5242C	K153T		TspEI	-2.5	0.00	EC144336 A	Homo
8	G C	A5242C	K153T		TspEI	-2.5	0.00	EC35272	Homo
9	G C	A5242C	K153T		TspEI	-2.5	0.00	EC433607	Homo
10	G C	A5242C	K153T		TspEI	-2.5	0.00	EC528362	Homo
11	G C	A5242C	K153T		TspEI	-2.5	0.00	EC1177297	Homo
12	G C	A5242C	K153T		TspEI	-2.5	0.00	EC6486	Homo
13	G C	A5242C	K153T		TspEI	-2.5	0.00	EC241446 A	Homo
14	G C	A5242C	K153T		TspEI	-2.5	0.00	IC469714	Homo
15	G C	A5242C	K153T		TspEI	-2.5	0.00	EC251649	Homo
16	G C	G5848A	R245Q		Hpy178III	-9.7	1.00	EC398716	Homo
17	G C	G5848A	R245Q		Hpy178III	-9.7	1.00	TLBR-2	Homo
18	G C	G5848A	R245Q		Hpy178III	-9.7	1.00	EC490128	Homo
19	G C	G5848A	R245Q		Hpy178III	-9.7	1.00	EC144336 A	Homo
20	G C	G5848A	R245Q		Hpy178III	-9.7	1.00	EC433607	Homo
21	G C	G5848A	R245Q		Hpy178III	-9.7	1.00	EC1177297	Homo
22	G C	G5848A	R245Q		Hpy178III	-9.7	1.00	EC241446 A	Homo
23	G C	G5848A	R245Q		Hpy178III	-9.7	1.00	EC251649	Homo
24	G C	G5848A	R245Q		Hpy178III	-9.7	1.00	EC529086	Homo
25	G C	G5848A	R245Q		Hpy178III	-9.7	1.00	EC35252	Homo
26	G C	G5848A	R245Q		Hpy178III	-9.7	1.00	EC528362	Homo
27	G C	G5848A	R245Q		Hpy178III	-9.7	1.00	IC469714	Homo
28	G C	G5848A	R245Q		Hpy178III	-9.7	1.00	EC398710	Homo
29	G C	G5848A	R245Q		Hpy178III	-9.7	1.00	EC6486	Homo
30	G C	G5848A	R245Q		Hpy178III	-9.7	1.00	EC35272	Homo
31	G C	A7210G	S377=	MnlI, TaqI				EC538139	Homo

**32**	G	T10319A	Intron					Feb.4	Homo
**33**	G	T10319A	Intron					LA3995	Homo
**34**	G	T10319A	Intron					AGATA-30	Homo
**35**	G	T10319A	Intron					Sel-14	Homo
**36**	G	T10319A	Intron					Castle Rock	Homo
**37**	G	G10331GG	Intron	TaqII				Castle Rock	Homo
**38**	G	G10331GG	Intron	TaqII				AGATA-30	Homo
**39**	G	G10331GG	Intron	TaqII				LA3995	Homo
**40**	G	G10331GG	Intron	TaqII				Sel-14	Homo
**41**	G	G10331GG	Intron	TaqII				Feb.4	Homo
**42**	G	T10332G	Intron		Bsp1407I, MjaIV, TatI			LA3995	Homo
**43**	G	T10332G	Intron		Bsp1407I, MjaIV, TatI			AGATA-30	Homo
**44**	G	T10332G	Intron		Bsp1407I, MjaIV, TatI			Sel-14	Homo
**45**	G	T10332G	Intron		Bsp1407I, MjaIV, TatI			Castle Rock	Homo
**46**	G	T10332G	Intron		Bsp1407I, MjaIV, TatI			Feb.4	Homo
**47**	G	C10339T	Intron					AGATA-30	Homo
**48**	G	C10339T	Intron					Feb.4	Homo
**49**	G	C10339T	Intron					Castle Rock	Homo
**50**	G	C10339T	Intron					LA3995	Homo
**51**	G	C10339T	Intron					Sel-14	Homo
**52**	G C	T10358G	V525=	BseSI, HgiJII, SduI				AGATA-30	Homo
**53**	G C	T10358G	V525=	BseSI, HgiJII, SduI				LA3995	Homo
**54**	G C	T10358G	V525=	BseSI, HgiJII, SduI				Sel-14	Homo
**55**	G C	T10358G	V525=	BseSI, HgiJII, SduI				Castle Rock	Homo
**56**	G C	T10358G	V525=	BseSI, HgiJII, SduI				Feb.4	Homo
**57**	G C	T10358G	V525=	BseSI, HgiJII, SduI				EC531801	Homo
**58**	G C	A10381AA	Frameshift	TspEI	MslI, TspDTI			Sel-14	Homo
**59**	G C	A10381AA	Frameshift	TspEI	MslI, TspDTI			AGATA-30	Homo
**60**	G C	A10381AA	Frameshift	TspEI	MslI, TspDTI			Castle Rock	Homo
**61**	G C	A10381AA	Frameshift	TspEI	MslI, TspDTI			Feb.4	Homo
**62**	G C	A10381AA	Frameshift	TspEI	MslI, TspDTI			LA3995	Homo
**63**	G C	A10408AA	Frameshift		Cac8I			Sel-14	Homo
**64**	G C	A10408AA	Frameshift		Cac8I			AGATA-30	Homo
**65**	G C	A10408AA	Frameshift		Cac8I			LA3995	Homo
**66**	G C	A10408AA	Frameshift		Cac8I			Feb.4	Homo
**67**	G C	A10408AA	Frameshift		Cac8I			Castle Rock	Homo
**68**	G	G10826A	Intron					Sel-14	Homo
**69**	G	G10826A	Intron					Feb.4	Homo
**70**	G	G10826A	Intron					LA3995	Homo
**71**	G	G10826A	Intron					AGATA-30	Homo
**72**	G	G10826A	Intron					Castle Rock	Homo
**73**	G	G10826A	Intron					EC531801	Homo
**74**	G	C10925CACC	Intron					Castle Rock	Homo
**75**	G	C10925CACC	Intron					AGATA-30	Homo
**76**	G	C10925CACC	Intron					Feb.4	Homo
**77**	G	C10925CACC	Intron					Sel-14	Homo
**78**	G	C10925CACC	Intron					LA3995	Homo

79	G	C10925CACC	Intron					EC531801	Homo
80	G C	G11077A	E639K			8.3	0.23	EC531801	Homo

CYC-B

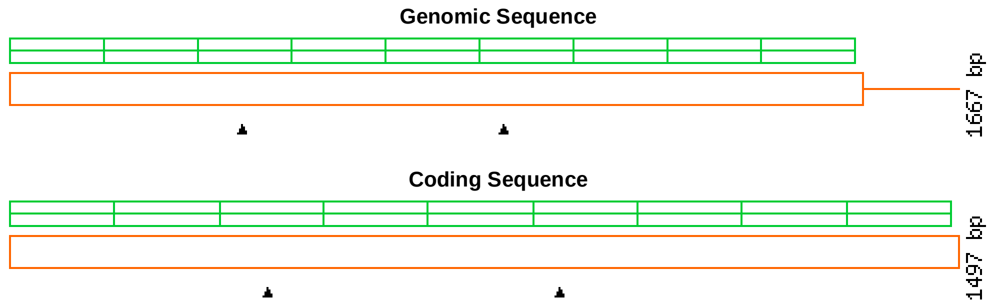

#	View On Sequence	Nucleotide Change	Effect	Restriction Enyzme Differences from REBASE		PSSM Difference	SIFT Score	Accession	Zygosity
				Gained in Variant	Lost from Reference				
1	G C	G406A	D136N	TspEI	MboII	2.1	0.00	LA0292	Homo
2	G C	G868A	D290N	ApoI, TspEI		-2.5	0.00	EC129602	Homo
3	G C	G868A	D290N	ApoI, TspEI		-2.5	0.00	LA0292	Homo

MSH2

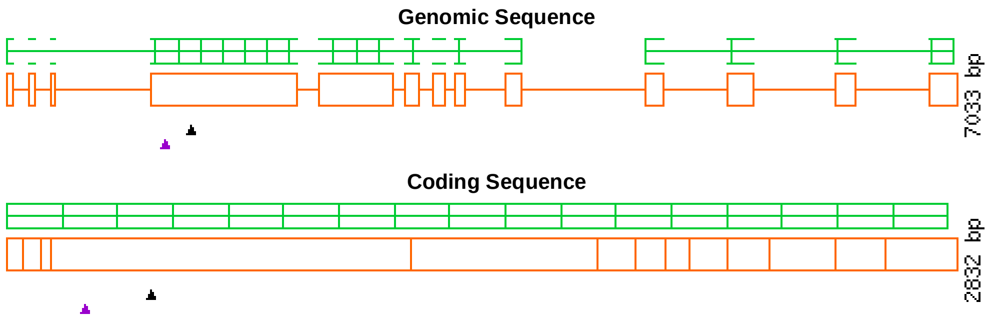

#	View On Sequence	Nucleotide Change	Effect	Restriction Enyzme Differences from REBASE		PSSM Difference	SIFT Score	Accession	Zygosity
				Gained in Variant	Lost from Reference				
1	G C	G1168T	A77=	BseSI, HgiAI, SduI	HhaI			EC398695	Homo
2	G C	T1365C	V143A	MwoI	BsrI, TspRI	0.5	0.00	EC398695	Homo

NAC-NOR

Genomic Sequence

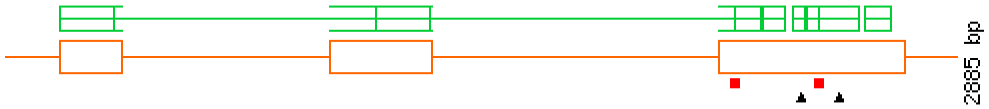

Coding Sequence

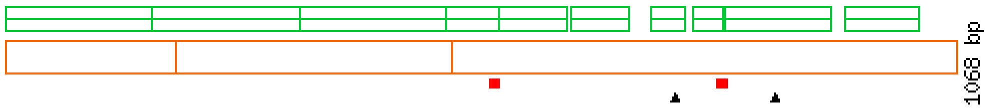

#	View On Sequence	Nucleotide Change	Effect	Restriction Enyzme Differences from REBASE		PSSM Difference	SIFT Score	Accession	Zygosity
				Gained in Variant	Lost from Reference				
1	G C	A2209:	Frameshift	TstI				LA3770	Homo
2	G C	A2210:	Frameshift	TstI				LA3770	Homo
3	G C	A2412T	T251S	ClaI, DpnI, MboI, TaqI		0.3	0.42	Feb.4	Homo
4	G C	A2465:	Frameshift					EC398712	Homo
5	G C	A2465:	Frameshift					EC398714	Homo
6	G C	A2465:	Frameshift					EC398716	Homo
7	G C	A2465:	Frameshift					EC398687	Homo
8	G C	A2465:	Frameshift					EC398697	Homo
9	G C	A2466:	Frameshift					EC398687	Homo
10	G C	A2466:	Frameshift					EC398716	Homo
11	G C	A2466:	Frameshift					EC398714	Homo
12	G C	A2466:	Frameshift					EC398697	Homo
13	G C	A2466:	Frameshift					EC398712	Homo
14	G C	C2467:	Frameshift	SspI				EC398687	Homo
15	G C	C2467:	Frameshift	SspI				EC398716	Homo
16	G C	C2467:	Frameshift	SspI				EC398697	Homo
17	G C	C2467:	Frameshift	SspI				EC398714	Homo
18	G C	C2467:	Frameshift	SspI				EC398714	Homo
19	G C	G2526A	D289N		BccI	-3.2	1.00	EC398712	Homo
20	G C	G2526A	D289N		BccI	-3.2	1.00	EC398697	Homo
21	G C	G2526A	D289N		BccI	-3.2	1.00	EC398714	Homo
22	G C	G2526A	D289N		BccI	-3.2	1.00	EC398716	Homo
23	G C	G2526A	D289N		BccI	-3.2	1.00	EC398687	Homo

PHOT1

Genomic Sequence

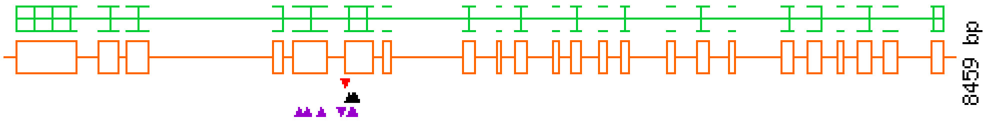

Coding Sequence

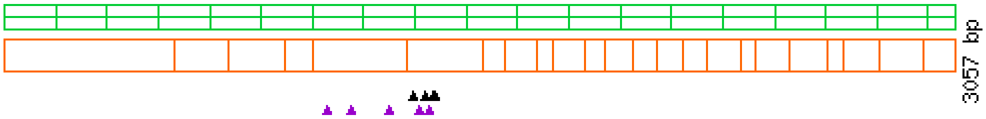

#	View On Sequence	Nucleotide Change	Effect	Restriction Enyzme Differences from REBASE		PSSM Difference	SIFT Score	Accession	Zygosity
				Gained in Variant	Lost from Reference				
1	G C	A2610G	L346=					TLBR-2	Homo
2	G C	A2688C	S372=					TLBR-2	Homo
3	G C	G2808A	K412=	MslI, TspEI	MboII			TLBR-2	Homo
4	G	T2997G	Intron		ApaBI, BsrDI, CviRI			TLBR-2	Homo
5	G	A3033G	Splice Junction		Bsp1407I, TatI			TLBR-2	Homo
6	G C	G3058T	S439I		MaeI, RsaI, ScaI, TatI	-1.6	0.00	TLBR-2	Homo
7	G C	T3074C	T444=					TLBR-2	Homo
8	G C	A3096G	R452G			0.1	0.00	TLBR-2	Homo
9	G C	A3104T	T454=					TLBR-2	Homo
10	G C	G3120C	D460H	DpnI, Hpy178III, MboI, MslI		-3.2	0.00	TLBR-2	Homo
11	G C	G3123T	D461Y			-1.5	0.00	TLBR-2	Homo

PHYA

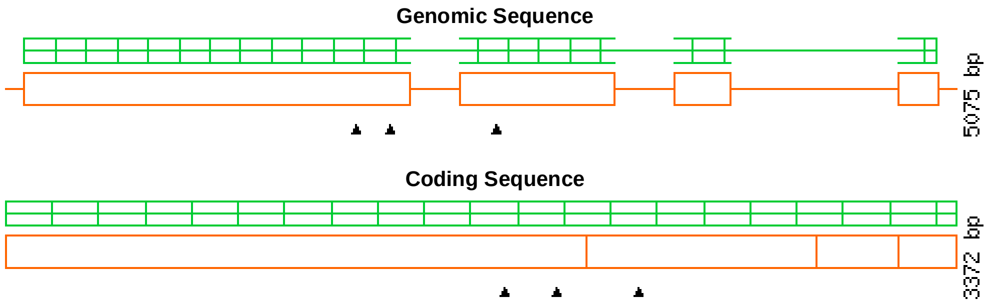

#	View On Sequence	Nucleotide Change	Effect	Restriction Enzyme Differences from REBASE		PSSM Difference	SIFT Score	Accession	Zygosity
				Gained in Variant	Lost from Reference				
1	G C	G1870T	E590D		BbvCI, Bpu10I, BseMII, DdeI, MnlI	0.9	0.00	EC521067 B	Homo
2	G C	A2053T	L651F	ApoI	MseI	-1.0	0.00	EC521067 B	Homo
3	G C	G2614A	E748K		TaqI	2.0	0.00	EC528362	Homo

PHYB1

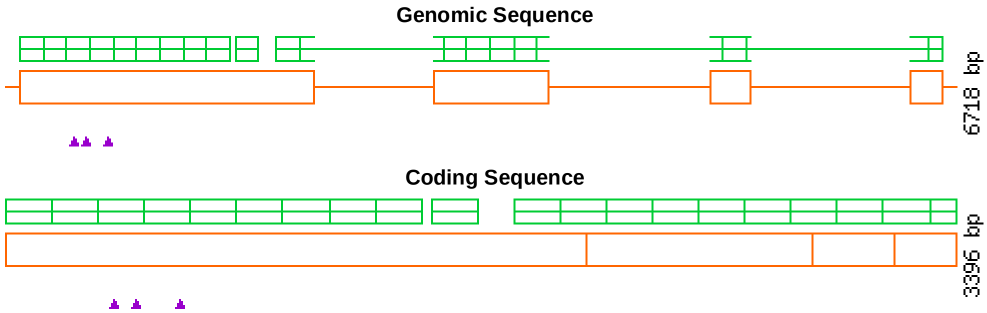

#	View On Sequence	Nucleotide Change	Effect	Restriction Enzyme Differences from REBASE		PSSM Difference	SIFT Score	Accession	Zygosity
				Gained in Variant	Lost from Reference				
1	G C	A484T	L128=		MaeI			EC521067 B	Homo
2	G C	C568T	F156=		BsmI			EC521067 B	Homo
3	G C	C724A	A208=	BtsI, TspRI	BcefI, CviJI, NlaIV			EC521067 B	Homo
