# Supplementary material for: Tomato Fruits Show Wide Phenomic Diversity but Fruit Developmental Genes Show Low Genomic Diversity
Source: PLoS One. 2016 Apr 14;11(4):e0152907. doi: 10.1371/journal.pone.0152907 (PMC4831840; doi:10.1371/journal.pone.0152907)
Supplement: S1 Table — Numbers 1–37 and 38–49 represent the shape and color variables digitally collected by TA. (DOCX) [file pone.0152907.s013.docx]

**S1 Table.** Communalities in Factor analysis: Proportion of variance explained by the extracted factors for each of the variables. Numbers 1-37 and 38-49 represent the shape and color variables digitally collected by TA.

|  | **Variables** | **Initial** | **Extraction** |  | **Variables** | **Initial** | **Extraction** |
| --- | --- | --- | --- | --- | --- | --- | --- |
| 1. | Perimeter | 1.000 | .982 | 26. | V Asymmetry | 1.000 | .814 |
| 2. | Area | 1.000 | .975 | 27. | H Asymmetry ob | 1.000 | .809 |
| 3. | Width Midheight | 1.000 | .985 | 28. | H Asymmetry ov | 1.000 | .910 |
| 4. | Maximum Width | 1.000 | .985 | 29. | Width Widest Pos | 1.000 | .716 |
| 5. | Height Midwidth | 1.000 | .978 | 30. | Eccentricity | 1.000 | .961 |
| 6. | Maximum Height | 1.000 | .981 | 31. | Proximal Eccentricity | 1.000 | .902 |
| 7. | Fruit Shape Index External1 | 1.000 | .967 | 32. | Distal Eccentricity | 1.000 | .909 |
| 8. | Fruit Shape Index External2 | 1.000 | .980 | 33. | Fruit Shape Index Internal | 1.000 | .959 |
| 9. | Proximal fruit blockiness | 1.000 | .748 | 34. | Eccentricity Area Index | 1.000 | .946 |
| 10. | Distal end fruit blockiness | 1.000 | .847 | 35. | Lobedness Degree | 1.000 | .894 |
| 11. | Fruit shape Triangle | 1.000 | .800 | 36. | Pericarp Area | 1.000 | .817 |
| 12. | Ellipsoid | 1.000 | .929 | 37. | Pericarp Thickness | 1.000 | .796 |
| 13. | Circular | 1.000 | .909 | 38. | Parameter1 | 1.000 | .503 |
| 14. | Rectangular | 1.000 | .899 | 39. | Parameter2 | 1.000 | .661 |
| 15. | Heart Shape | 1.000 | .435 | 40. | Avg Red | 1.000 | .982 |
| 16. | Shoulder Height | 1.000 | .892 | 41. | Avg Green | 1.000 | .987 |
| 17. | Proximal Angle Micro | 1.000 | .748 | 42. | Avg Blue | 1.000 | .937 |
| 18. | Proximal Angle Macro | 1.000 | .772 | 43. | Avg Luminosity | 1.000 | .986 |
| 19. | Proximal Indentation Area | 1.000 | .922 | 44. | Avg L | 1.000 | .996 |
| 20. | Distal Angle Micro | 1.000 | .498 | 45. | Avg a* | 1.000 | .955 |
| 21. | Distal Angle Macro | 1.000 | .875 | 46. | Avg b* | 1.000 | .952 |
| 22. | Distal Indentation Area | 1.000 | .702 | 47. | Avg Hue | 1.000 | .975 |
| 23. | Distal End Protrusion | 1.000 | .670 | 48. | Avg Chroma | 1.000 | .889 |
| 24. | Obovoid | 1.000 | .827 | 49. | a*/b* | 1.000 | .981 |
| 25. | Ovoid | 1.000 | .891 |  |  |  |  |
