# Supplementary material for: Tomato Fruits Show Wide Phenomic Diversity but Fruit Developmental Genes Show Low Genomic Diversity
Source: PLoS One. 2016 Apr 14;11(4):e0152907. doi: 10.1371/journal.pone.0152907 (PMC4831840; doi:10.1371/journal.pone.0152907)
Supplement: S2 Table — (DOCX) [file pone.0152907.s014.docx]

**S2 Table.** Categories of phenotypic characters and their percentage in the population.

|  |  | **Category** | **Sub-category** | **No. of** | **Accessions** | **%** |  |
| --- | --- | --- | --- | --- | --- | --- | --- |
|  |  |  |  | **accessions** | **showing** | **variation** |  |
|  |  |  |  |  | **variation** |  |  |
| **I** | **Architecture** | **1. Plant size** | Short (< 90 cm) | 5 | 8 | 6.30 |  |
|  |  |  |  |  |  |  |  |
|  |  |  | Medium (90-100 | 119 |  |  |  |
|  |  |  | cm) |  |  |  |  |
|  |  |  | Tall (> 100 cm) | 3 |  |  |  |
|  |  |  |  |  |  |  |  |
|  |  | **2. Branching/other growth pattern** | Normal branched | 41 | 86 | 67.72 |  |
|  |  |  | (5-8 Branches) |  |  |  |  |
|  |  |  | More branched (>8 | 84 |  |  |  |
|  |  |  | branches) |  |  |  |  |
|  |  |  | Aborted growth | 1 |  |  |  |
|  |  |  |  |  |  |  |  |
|  |  |  | Other plant habit | 1 |  |  |  |
|  |  |  |  |  |  |  |  |
|  |  | **3. Internodal** | Short (< 6 cm) | 5 | 8 | 6.30 |  |
|  |  | **length** |  |  |  |  |  |
|  |  |  | Medium (6-10cm) | 119 |  |  |  |
|  |  |  |  |  |  |  |  |
|  |  |  |  |  |  |  |  |
|  |  |  | Long (> 6cm) | 3 |  |  |  |
|  |  |  |  |  |  |  |  |
| **II** | **Leaf** | **4. Leaf color** | Dark green | 114 | 13 | 10.24 |  |
|  |  |  |  |  |  |  |  |
|  |  |  | Very dark green | 9 |  |  |  |
|  |  |  |  |  |  |  |  |
|  |  |  | Yellowish green | 3 |  |  |  |
|  |  |  |  |  |  |  |  |
|  |  |  | Whitish green | 1 |  |  |  |
|  |  |  |  |  |  |  |  |
|  |  | **5. Leaf width** | Short (< 6 cm) | 8 | 8 | 6.30 |  |
|  |  |  |  |  |  |  |  |
|  |  |  | Medium (6-10cm) | 119 |  |  |  |
|  |  |  |  |  |  |  |  |
|  |  | **6. Leaf size** | Small (<5 cm) | 12 | 13 | 10.24 |  |
|  |  |  |  |  |  |  |  |
|  |  |  | Medium (5-10 cm) | 114 |  |  |  |
|  |  |  |  |  |  |  |  |
|  |  |  | Large (>10 cm) | 1 |  |  |  |
|  |  |  |  |  |  |  |  |
|  |  | **7. Leaf** | Simple | 2 | 6 | 4.72 |  |
|  |  | **complexity** |  |  |  |  |  |
|  |  |  | Intermediate | 4 |  |  |  |
|  |  |  |  |  |  |  |  |
|  |  |  |  |  |  |  |  |
|  |  |  | Compound | 121 |  |  |  |
|  |  |  |  |  |  |  |  |
|  |  | **8. Leaf texture** | Rough | 80 | 47 | 37.01 |  |
|  |  |  |  |  |  |  |  |
|  |  |  | Very rough | 27 |  |  |  |
|  |  |  |  |  |  |  |  |
|  |  |  | Smooth | 16 |  |  |  |
|  |  |  |  |  |  |  |  |
|  |  |  | Thick | 4 |  |  |  |
|  |  |  |  |  |  |  |  |
|  |  | **9. Other Leaf** | No edge | 1 | 9 | 7.09 |  |
|  |  | **Developments** | Plane leaf edge | 1 |  |  |  |
|  |  |  | Small leaflets | 3 |  |  |  |
|  |  |  | Variegated yellow | 2 |  |  |  |
|  |  |  | and green leaves |  |  |  |  |
|  |  |  | Wilted leaf | 2 |  |  |  |
| **III** | **Inflorescence** | **10. Inflorescence** | Small (< 8cm) | 4 | 4 | 3.15 |  |
|  |  |  |  |  |  |  |  |
|  |  |  | Medium (8-12 cm) | 123 |  |  |  |
|  |  |  |  |  |  |  |  |
| **IV** | **Flower** | **11. Flower** | Small flower | 2 | 6 | 4.72 |  |
|  |  | **morphology** |  |  |  |  |  |
|  |  |  | Normal flower | 121 |  |  |  |
|  |  |  |  |  |  |  |  |
|  |  |  |  |  |  |  |  |

|  |  | **Category** | **Sub-category** | **No. of** | **Accessions** | **%** |  |
| --- | --- | --- | --- | --- | --- | --- | --- |
|  |  |  |  | **accessions** | **showing** | **variation** |  |
|  |  |  |  |  | **variation** |  |  |
|  |  |  | Altered floral | 1 |  |  |  |
|  |  |  | organ size |  |  |  |  |
|  |  |  | Broad petal | 1 |  |  |  |
|  |  |  |  |  |  |  |  |
|  |  |  | Other | 2 |  |  |  |
|  |  |  |  |  |  |  |  |
|  |  | **12. Flower color** | Yellow | 94 | 33 | 25.98 |  |
|  |  |  |  |  |  |  |  |
|  |  |  | Pale yellow | 29 |  |  |  |
|  |  |  |  |  |  |  |  |
|  |  |  | Strong yellow | 4 |  |  |  |
|  |  |  |  |  |  |  |  |
| **V** | **Fruit** | **13. Fruit size** | Small | 15 | 31 | 24.41 |  |
|  |  |  |  |  |  |  |  |
|  |  |  | Medium | 96 |  |  |  |
|  |  |  |  |  |  |  |  |
|  |  |  | Large | 16 |  |  |  |
|  |  |  |  |  |  |  |  |
|  |  | **14. Fruit** | Round | 104 | 23 | 18.11 |  |
|  |  | **morphology** |  |  |  |  |  |
|  |  |  | Deformed fruit | 1 |  |  |  |
|  |  |  |  |  |  |  |  |
|  |  |  |  |  |  |  |  |
|  |  |  | Fused | 1 |  |  |  |
|  |  |  |  |  |  |  |  |
|  |  |  | Hairy stripe | 1 |  |  |  |
|  |  |  |  |  |  |  |  |
|  |  |  | Long | 7 |  |  |  |
|  |  |  |  |  |  |  |  |
|  |  |  | Notched | 1 |  |  |  |
|  |  |  |  |  |  |  |  |
|  |  |  | Oval | 5 |  |  |  |
|  |  |  |  |  |  |  |  |
|  |  |  | Pumpkin type | 6 |  |  |  |
|  |  |  |  |  |  |  |  |
|  |  |  | Other | 1 |  |  |  |
|  |  |  |  |  |  |  |  |
|  |  | **15. Fruit color** | Red | 94 | 31 | 25.98 |  |
|  |  |  |  |  |  |  |  |
|  |  |  | Dark red | 20 |  |  |  |
|  |  |  |  |  |  |  |  |
|  |  |  | Light pigmented | 1 |  |  |  |
|  |  |  |  |  |  |  |  |
|  |  |  | Orange | 2 |  |  |  |
|  |  |  |  |  |  |  |  |
|  |  |  | Pink | 7 |  |  |  |
|  |  |  |  |  |  |  |  |
|  |  |  | Yellow | 0 |  |  |  |
|  |  |  |  |  |  |  |  |
|  |  |  | Striped | 1 |  |  |  |
|  |  |  |  |  |  |  |  |
|  |  |  | Others | 2 |  |  |  |
|  |  |  |  |  |  |  |  |
|  |  | **16. Ripening** | Normal (45-48 | 123 | 4 | 3.15 |  |
|  |  |  | days post anthesis |  |  |  |  |
|  |  |  | for reaching red |  |  |  |  |
|  |  |  | ripe stage) |  |  |  |  |
|  |  |  | Late (>50 days) | 4 |  |  |  |
|  |  |  |  |  |  |  |  |
