# Supplementary material for: Tomato Fruits Show Wide Phenomic Diversity but Fruit Developmental Genes Show Low Genomic Diversity
Source: PLoS One. 2016 Apr 14;11(4):e0152907. doi: 10.1371/journal.pone.0152907 (PMC4831840; doi:10.1371/journal.pone.0152907)
Supplement: S3 Table — (DOCX) [file pone.0152907.s015.docx]

**S3 Table.** List of primers used for screening of SNPs.

|  |  | **Sequence** | |  |
| --- | --- | --- | --- | --- |
|  | **Primer** |  |  |  |
|  |  | **Forward** | **Reverse** |  |
|  | **Name** |  |  |  |
|  | *ACS2.1* | 5´-GACCATTGCTTATCGAGGTAAAAT-3´ | 5´-CGAAAGTCGATTCCCTTAAAAGT-3´ |  |
|  | *ACS2.2* | 5´-TTAGCGGCAATGCTATCGGA-3´ | 5´-CACAAACACCATAATCTCTCCATCTC-3´ |  |
|  | *COP1.3* | 5´-TACAGCTCGAACAATCACGACCTT-3´ | 5´-GCATTGACCAGCAGTTCAGAAGTG-3´ |  |
|  | *COP1.4* | 5´-CTCAAGTTCTTTGTGCTTCAACCCA-3´ | 5´-ACCAAGCCCTACAACTTGTGTTAC-3´ |  |
|  | *CYC-B* | 5´-CTCTTCTCAAGCCTTTTCCATCTC-3´ | 5´-CTAACACATCTTCTATCCAAAGGC-3´ |  |
|  | *PHYA.1* | 5´-ACACGTCAACAAGGAACTGGAATTGGAAAATC-3´ | 5´-CTGATCAATTTGGCTGGTGTTCTGAGTGGA-3´ |  |
|  | *PHYA.2* | 5´-AGGTAGAGGCTTTACGATAAATCATCC-3´ | 5´-AATTCCAGTTCCTTGTTGACGTGTATG-3´ |  |
|  | *PHYB1* | 5´-CATCACAAGGTCAAGCTCAATCTTCAGG-3´ | 5´-TCCACAATCATTCTCACCCTGTCCTGC-3´ |  |
|  | *PHOT1* | 5´-AAACTTCTGACTCGGCTATG-3´ | 5´-TTCTTCCCAAGATTTCTTCA-3´ |  |
|  | *MSH2* | 5´-TGTACCAATGTGCATTTTCTTCTT-3´ | 5´-AGCTAAGAAAAGAGGGGATTCAA-3´ |  |
|  | *NAC-NOR* | 5´-ACTTAGTGGTTTCAGGGGTTCA-3´ | 5´-CCAATTCATGCCAGTAACTTGA-3´ |  |
|  | *PSY1.1* | 5´-TTGTTATGGGTTGTTTCTCCTTGTGAC-3´ | 5´-TAGGACGAGAGAAACAGATATAGGAGACAG-3´ |  |
|  | *PSY1.2* | 5´-AGGTGGGAAAATAGGCTAGAAGAT-3´ | 5´-GCACTTCCAAAGAAAGAATAAAGG-3´ |  |
|  | *PSY1.3* | 5´-ATACCTGTCTAGTCGGCGTTTATC-3´ | 5´-TGTAGTCATTGGCTTCAATCTCAT-3´ |  |
|  | *RIN2* | 5’-GAAACTCACGAAATTACGACAA-3’ | 5’-ATATGATCGATTACTGAACTTTA-3’ |  |
|  | *RIN3* | 5’-TGTCTCACTAAGGTAACTTCATTATCA-3’ | 5’-CAATATTGCCATACTCTTCTTGACA-3’ |  |
|  | *M13* | 5’-IRD_700_-TGTAAAACGACGGCCAGT-3’ | 5’-IRD_800_-AGGAAACAGCTATGACCAT-3’ |  |
