# Supplementary material for: Tomato Fruits Show Wide Phenomic Diversity but Fruit Developmental Genes Show Low Genomic Diversity
Source: PLoS One. 2016 Apr 14;11(4):e0152907. doi: 10.1371/journal.pone.0152907 (PMC4831840; doi:10.1371/journal.pone.0152907)
Supplement: S4 Table — (DOCX) [file pone.0152907.s016.docx]

**S4 Table.** List of accessions used in the study.

| **S. No** | **Accessions** | **Details** | | | **Biological status** | **Source** |
| --- | --- | --- | --- | --- | --- | --- |
| 1. | Arka Vikas | Sel22/ Arka Vikas | | | Released | Karnataka, |
|  |  |  | | | variety | India |
| 2. | UI.1 | Unidentified | | | - | Unknown, |
|  |  |  | | |  | exotic |
| 3. | EC 241446 A | M-2495 | | | Landrace | Israel |
|  |  |  | | |  |  |
| 4. | EC 251649 | LO1033 | | | Landrace | Taiwan |
|  |  |  | | |  |  |
| 5. | EC 320583 | Veepick VF / 419A | | | Landrace | Canada |
|  |  |  | | |  |  |
| 6. | EC 315478 | NC-2C | | | Landrace | USA |
|  |  |  | | |  |  |
| 7. | EC 339066 | CL 5915-93D4-1-0-3 | | | Landrace | Taiwan |
|  |  |  | | |  |  |
| 8. | EC 338717 | CL 5915-93 D4-1-0-12 | | | Landrace | Taiwan |
|  |  |  | | |  |  |
| 9. | EC 362941 | BL 342 / CRA 84-79,5 | | | Landrace | Taiwan |
|  |  |  | | |  |  |
| 10. | EC 362949 | CL 1131-0-0-13-0-6/L1 (VC48-1GS) /L283 | | | Landrace | Taiwan |
|  |  | (Tamu Chico III) 200gm 1ut L 127 (Tm-2a) / | | |  |  |
|  |  | L21 (VC) | | |  |  |
| 11. | UI.2 | Unidentified | | | - | Unknown, |
|  |  |  | | |  | exotic |
| 12. | EC 369020 | 268 | | | Landrace | Italy |
|  |  |  | | |  |  |
| 13. | UI.3 | Unidentified | | | - | Unknown, |
|  |  |  | | |  | exotic |
| 14. | EC 398684 | - | | | Variety | Australia |
|  |  |  | | |  |  |
| 15. | EC 398685 | - | | | Variety | Australia |
|  |  |  | | |  |  |
| 16. | EC 398688 | - | | | Variety | Australia |
|  |  |  | | |  |  |
| 17. | EC 398687 | - | | | Variety | Australia |
|  |  |  | | |  |  |
| 18. | EC 398695 | - | | | Variety | Australia |
|  |  |  | | |  |  |
| 19. | EC 398697 | - | | | Variety | Australia |
|  |  |  | | |  |  |
| 20. | EC 398704 | - | | | Variety | Australia |
|  |  |  | | |  |  |
| 21. | EC 398710 | - | | | Variety | Australia |
|  |  |  | | |  |  |
| 22. | EC 398712 | - | | | Variety | Australia |
|  |  |  | | |  |  |
| 23. | EC 398714 | - | | | Variety | Australia |
|  |  |  | | |  |  |
| 24. | EC 398716 | - | | | Variety | Australia |
|  |  |  | | |  |  |
| 25. | EC 398717 | - | | | Variety | Australia |
|  |  |  | | |  |  |
| 26. | UI.4 | Unidentified | | | - | Unknown, |
|  |  |  | | |  | exotic |
| 27. | UI.5 | Unidentified | | | - | Unknown, |
|  |  |  | | |  | exotic |
| 28. | EC-6875 | Station Red Pear | | | Landrace | Unknown, |
|  |  |  | | |  | exotic |
| 29. | EC-7317 | C.S.I.R.O. NO. 12 HETTNERSx H.E.S. | | | Landrace | Australia |
|  |  | 4242xAustralian Dwarf Red | | |  |  |
| 30. | UI.6 | Unidentified | | | - | Unknown, |
|  |  |  | | |  | exotic |
| 31. | EC-13274 | 41.2504B | | | Landrace | USA |
|  |  |  | | |  |  |
| 32. | EC-13574 | Morada | |  | Landrace | Venezuela |
|  |  |  | |  |  |  |
| 33. | EC-16343 | 106/26 | |  | Landrace | Italy |
|  |  |  | |  |  |  |
| 34. | EC-27885 | White | |  | Landrace | Ghana |
|  |  |  | |  |  |  |
| 35. | UI.7 | Unidentified | |  | - | Unknown, |
|  |  |  | |  |  | exotic |
| 36. | UI.8 | Unidentified | |  | - | Unknown, exotic |
| 37. | UI.9 | Unknown | |  | Landrace | Unknown, |
|  |  |  | |  |  | exotic |
| 38. | EC-35252 | Kronekamp | |  | Landrace | Hungary |
|  |  |  | |  |  |  |
| 39. | EC-35272 | Tschudo Rinka | |  | Landrace | Hungary |
|  |  |  | |  |  |  |
| 40. | EC-35322 | Abd | |  | Landrace | Hungary |
|  |  |  | |  |  |  |
| 41. | UI.10 | Unidentified | |  | - | Unknown, |
|  |  |  | |  |  | exotic |
| 42. | EC-129602 | Petomech | |  | Landrace | New Zealand |
|  |  |  | |  |  |  |
| 43. | EC-129604 | - | |  | Variety | New Zealand |
|  |  |  | |  |  |  |
| 44. | UI.11 | Unidentified | |  | - | Unknown, |
|  |  |  | |  |  | exotic |
| 45. | IC 447708 | - | |  | Improved | Himachal |
|  |  |  | |  | Cultivar | Pradesh, |
|  |  |  | |  |  | India |
| 46. | EC 357828 | BL 350 | |  | Landrace | Unknown, |
|  |  |  | |  |  | exotic |
| 47. | UI.12 | Unidentified | |  | - | Unknown, |
|  |  |  | |  |  | exotic |
| 48. | EC 490128 | - | |  | Breeding | Taiwan |
|  |  |  | |  | lines |  |
| 49. | EC 490130 | - | |  | Breeding | Taiwan |
|  |  |  | |  | lines |  |
| 50. | IC 469648 | JSR/04-54 | |  | - | Tumkur, |
|  |  |  | |  |  | India |
| 51. | IC 469628 | JSR/04-34 | |  | - | Tumkur, |
|  |  |  | |  |  | India |
| 52. | IC 469626 | JSR/04-32 | |  | - | Tumkur, |
|  |  |  | |  |  | India |
| 53. | IC 469603 | JSR/04-09 | |  | - | Tumkur, |
|  |  |  | |  |  | India |
| 54. | EC 57442 | Strong Acid # 49 | |  | Landrace | Canada |
|  |  |  | |  |  |  |
| 55. | IC 469597 | Natti | |  | Traditional | Karnataka, |
|  |  |  | |  | or Farmers | India |
|  |  |  | |  | Variety |  |
| 56. | IC 469714 | JSR/04-120 | |  | - | Uttara |
|  |  |  | |  |  | Kannada, |
|  |  |  | |  |  | India |
| 57. | EC531801 | - | |  | Breeding | Taiwan |
|  |  |  | |  | lines |  |
| 58. | EC 529086 | - | |  | Breeding | China |
|  |  |  | |  | lines |  |
| 59. | EC 531802 | - | |  | Breeding | Taiwan |
|  |  |  | |  | lines |  |
| 60. | EC 398710 | - | |  | Variety | Australia |
|  |  |  | |  |  |  |
| 61. | EC 6486 | 127-91 |  | | Landrace | Egypt |
|  |  | HOPExTHATCHERxHINDI62x4/W.307 | | |  |  |
| 62. | EC 521067 B | PI-118325 |  | | Landrace | USA |
|  |  |  |  | |  |  |
| 63. | EC 521068 | - |  | | Variety | USA |
|  |  |  |  | |  |  |
| 64. | EC 538139 | - |  | | Variety | Russian |
|  |  |  |  | |  | Federation |
| 65. | EC 528362 | - |  | | Breeding | China |
|  |  |  |  | | Lines |  |
| 66. | WIR-13717 | *Lycopersicon* | *cerasiforme* accession now | |  | Unknown |
|  |  | named as *S. lycopersicum var cerasiforme* | | |  |  |
| 67. | EC- 8372 |  |  | |  | Unknown |
|  |  |  |  | |  |  |
| 68. | Pusa Rohini | Pusa Rohini |  | | Released | India |
|  |  |  |  | | variety |  |
| 69. | TLBR3 |  |  | | Genetic | Others |
|  |  |  |  | | Stock |  |
| 70. | PDT-3-1 |  |  | |  | IIVR, India |
|  |  |  |  | |  |  |
| 71. | TLBR2 |  |  | | Genetic | Others |
|  |  |  |  | | Stock |  |
| 72. | Rio Grande |  |  | | Landrace | Unknown, |
|  |  |  |  | |  | exotic |
| 73. | Castle Rock |  |  | |  | Unknown, |
|  |  |  |  | |  | exotic |
| 74. | KT-15 |  |  | |  | IIVR, India |
|  |  |  |  | |  |  |
| 75. | H-88-87 |  |  | |  | IIVR, India |
|  |  |  | | |  |  |
| 76. | *WIR-3928* | *Solanum corneliomuelleri* (formerly *Lycopersicon* | | |  | Unknown |
|  |  | *glandulosum*) |  | |  |  |
| 77. | EC-50-50 |  |  | |  | Unknown |
|  |  |  |  | |  |  |
| 78. | EC-3414425 |  |  | |  | Unknown |
|  |  |  | | |  |  |
| 79. | LA3995 | IL line (chromosome11) of *L. hirsutum* in the | | |  | TGRC, USA |
|  |  | background of *S. lycopersicum* | | |  |  |
| 80. | F-5070 |  |  | |  | IIVR, India |
|  |  |  | | |  |  |
| 81. | LA3967 | IL line (chromosome 11) of *L. hirsutum* in the | | |  | TGRC, USA |
|  |  | background of *S. lycopersicum* | | |  |  |
| 82. | LA3971 | IL line (chromosome 11) of *L. hirsutum* in the | | |  | TGRC, USA |
|  |  | background of *S. lycopersicum* | | |  |  |
| 83. | LA4040 | IL line (chromosome 2) of *L. pennellii* in the | | |  | TGRC, USA |
|  |  | background of *S. lycopersicum* | | |  |  |
| 84. | LA3934 | IL line (chromosome 4) of *L. hirsutum* in the | | |  | TGRC, USA |
|  |  | background of *S. lycopersicum* | | |  |  |
| 85. | Agata-30 | Introduction |  | |  | Russia |
|  |  |  |  | |  |  |
| 86. | BL-1208 |  |  | |  | IIVR, India |
|  |  |  |  | |  |  |
| 87. | TLBR12 |  |  | |  | IIVR, India |
|  |  |  | | |  |  |
| 88. | *L. esculentum* | *L. esculentum var. cerasiforme* | | |  | Unknown |
|  | *var. cerasiforme* | *Now named as S. lycopersicum var. cerasiforme* | | |  |  |
|  |  |  |  | |  |  |
| 89. | Palam Pink | Palam Pink |  | | Released | Himachal |
|  |  |  |  | | cultivar | Pradesh, |
|  |  |  |  | |  | India |
| 90. | IIHR-2201 |  | | | Genetic | Others |
|  |  |  | | | Stock |  |
| 91. | Vaibhav |  | | |  | IIVR, India |
|  |  |  | | |  |  |
| 92. | FEB4 |  | | |  | IIVR, India |
|  |  |  | | |  |  |
| 93. | Sel-14 |  | | |  | IIVR, India |
|  |  |  | | |  |  |
| 94. | LA3538 | *hp-1* (high pigment-1), *S. lycopersicum*, Ailsa | | |  | TGRC, USA |
|  |  | Craig | | |  |  |
| 95. | LA3530 | *gs* (green stripe), *S. lycopersicum*, Ailsa Craig | | |  | TGRC, USA |
|  |  |  | | |  |  |
| 96. | LA3539 | *ug* (uniform grey green), *S. lycopersicum*, Ailsa | | |  | TGRC, USA |
|  |  | Craig | | |  |  |
| 97. | LA3770 | *nor* (Non ripening), *S. lycopersicum* , Ailsa | | |  | TGRC, USA |
|  |  | Craig | | |  |  |
| 98. | LA3537 | *Nr* (Never ripe), *S. lycopersicum* | | |  | TGRC, USA |
|  |  |  | | |  |  |
| 99. | LA4025 | *Bog (Beta old gold***),** ***S. lycopersicum*** | | |  | TGRC, USA |
|  |  |  | | |  |  |
| 100. | LA1795 | *rin* (ripening inhibitor), *S. lycopersicum* | | |  | TGRC, USA |
|  |  |  | | |  |  |
| 101. | LA0292 | *Od*, *S. lycopersicum* var. cerasiforme | | |  | TGRC, USA |
|  |  |  | | |  |  |
| 102. | LA0276 | *S. lycopersicum* Red Top VF Ailsa Craig | | |  | TGRC, USA |
|  |  |  | | |  |  |
| 103. | LA3203 | *S. lycopersicum* cv. Large Plum | | |  | TGRC, USA |
|  |  |  | | |  |  |
| 104. | LA2818 | *S. lycopersicum* cv. Monalbo | | |  | TGRC, USA |
|  |  |  | | |  |  |
| 105. | LA2715 | *S. lycopersicum* cv. Porphyre | | |  | TGRC, USA |
|  |  |  | | |  |  |
| 106. | LA3632 | *S. lycopersicum* cv. Start 24 | | |  | TGRC, USA |
|  |  |  | | |  |  |
| 107. | LA1506 | *S. lycopersicum* cv. Stone | | |  | TGRC, USA |
|  |  |  | | |  |  |
| 108. | LA0266 | *S. lycopersicum* cv. Earli Pak | | |  | TGRC, USA |
|  |  |  | | |  |  |
| 109. | LA3231 | *S. lycopersicum* cv. Gulf State Market | | |  | TGRC, USA |
|  |  |  | | |  |  |
| 110. | LA3903 | *S. lycopersicum* cv. Prima Bel | | |  | TGRC, USA |
|  |  |  | | |  |  |
| 111. | LA3229 | *S. lycopersicum* cv. Prospero | | |  | TGRC, USA |
|  |  |  | | |  |  |
| 112. | LA1021 | *S. lycopersicum* cv. Santa Cruz | | |  | TGRC, USA |
|  |  |  | | |  |  |
| 113. | LA3237 | *S. lycopersicum* Homestead 24 | | |  | TGRC, USA |
|  |  |  | | |  |  |
| 114. | LA3234 | *S. lycopersicum* Sioux | | |  | TGRC, USA |
|  |  |  | | |  |  |
| 115. | LA3243 | *S. lycopersicum* Platense | | |  | TGRC, USA |
|  |  |  | | |  |  |
| 116. | LA1090 | *S. lycopersicum* Rutgers | | |  | TGRC, USA |
|  |  |  | | |  |  |
| 117. | LA2400 | *sp u S. lycopersicum* Cv. Castlemart | | |  | TGRC, USA |
|  |  |  | | |  |  |
| 118. | LA0274 | *d l w S. lycopersicum* | | |  | TGRC, USA |
|  |  |  | | |  |  |
| 119. | LA3554 | *yv S. lycopersicum* | | |  | TGRC, USA |
|  |  |  | | |  |  |
| 120. | LA0854 | *fa S. lycopersicum* | | |  | TGRC, USA |
|  |  |  | | |  |  |
| 121. | LA0215 | *at u y S. lycopersicum* | | |  | TGRC, USA |
|  |  |  | | |  |  |
| 122. | LA1016 | *dps S. lycopersicum* | | |  | TGRC, USA |
|  |  |  | | |  |  |
| 123. | LA2999 | *gf S. lycopersicum* | | |  | TGRC, USA |
|  |  |  | | |  |  |
| 124. | LA3430 | Xa-3 *S. lycopersicum* | | |  | TGRC, USA |
|  |  |  | | |  |  |
| 125. | LA2921 | *Del S. lycopersicum* | | |  | TGRC, USA |
|  |  |  | | |  |  |
| 126. | LA1996 | *Aft S. lycopersicum* | | |  | TGRC, USA |
|  |  |  | | |  |  |
| 127. | LA0744 | *S. lycopersicum* cv. VF11 | | |  | TGRC, USA |
