# Supplementary material for: Tomato Fruits Show Wide Phenomic Diversity but Fruit Developmental Genes Show Low Genomic Diversity
Source: PLoS One. 2016 Apr 14;11(4):e0152907. doi: 10.1371/journal.pone.0152907 (PMC4831840; doi:10.1371/journal.pone.0152907)
Supplement: S5 Table — The classes were obtained from agglomerative hierarchical clustering (by UPGMA method) of 127 accessions based on 55 fruit attributes. (DOCX) [file pone.0152907.s017.docx]

**S5 Table**. Performance analysis of 9 classes for 6 six fruit parameters based on class centroids. The classes were obtained from agglomerative hierarchical clustering (by UPGMA method) of 127 accessions based on 55 fruit attributes

| **Class** | **Weight**  **of fruit**  **(g)** | **V/H**  **Diameter**  **(mm)** | **pH** | **°Brix** | **Total carotenoid (μg/g FW)** | **a*/b*** |
| --- | --- | --- | --- | --- | --- | --- |
| **1** | 91.989 | 0.88 | 4.536 | 3.712 | 54.133 | 0.355 |
| **2** | 19.689 | 0.98 | 4.542 | 4.698 | 64.362 | 0.247 |
| **3** | 4.000 | 0.98 | 4.900 | 5.000 | 156.982 | 0.206 |
| **4** | 10.790 | 0.98 | 4.846 | 4.455 | 74.342 | 0.265 |
| **5** | 41.431 | 0.94 | 4.532 | 4.532 | 73.326 | 0.346 |
| **6** | 19.945 | 0.87 | 4.288 | 3.650 | 70.484 | 0.210 |
| **7** | 34.820 | 0.92 | 4.620 | 3.940 | 59.275 | 0.232 |
| **8** | 16.333 | 0.95 | 4.780 | 3.767 | 41.020 | 0.284 |
| **9** | 334.000 | 0.80 | 4.500 | 4.400 | 52.528 | 0.469 |
