# Supplementary material for: Tomato Fruits Show Wide Phenomic Diversity but Fruit Developmental Genes Show Low Genomic Diversity
Source: PLoS One. 2016 Apr 14;11(4):e0152907. doi: 10.1371/journal.pone.0152907 (PMC4831840; doi:10.1371/journal.pone.0152907)
Supplement: S6 Table — (DOCX) [file pone.0152907.s018.docx]

**S6 Table.** Total variance as explained by extracted factors in factor analysis.

|  | **Component** | **Eigenvalues** | **Rotation Sums of Squared Loadings** | |  |
| --- | --- | --- | --- | --- | --- |
|  |  |  | **% of Variance** | **Cumulative %** |  |
|  |  |  |  |  |  |
|  |  |  |  |  |  |
|  | 1 | 11.612 | 14.444 | 14.444 |  |
|  | 2 | 7.589 | 11.940 | 26.384 |  |
|  | 3 | 5.535 | 10.601 | 36.985 |  |
|  | 4 | 4.200 | 9.862 | 46.847 |  |
|  | 5 | 2.785 | 8.090 | 54.937 |  |
|  | 6 | 2.255 | 7.875 | 62.813 |  |
|  | 7 | 2.129 | 6.627 | 69.439 |  |
|  | 8 | 1.523 | 4.350 | 73.789 |  |
|  | 9 | 1.407 | 3.935 | 77.723 |  |
|  | 10 | 1.203 | 3.138 | 80.862 |  |
|  | 11 | 1.119 | 3.031 | 83.893 |  |
|  | 12 | 1.076 | 2.706 | 86.599 |  |
