# Supplementary material for: Tomato Fruits Show Wide Phenomic Diversity but Fruit Developmental Genes Show Low Genomic Diversity
Source: PLoS One. 2016 Apr 14;11(4):e0152907. doi: 10.1371/journal.pone.0152907 (PMC4831840; doi:10.1371/journal.pone.0152907)
Supplement: S7 Table — Variables Entered/Removed in the stepwise discriminant analysis of tomato shape (A). Summary of canonical discriminant functions- Eigenvalues (B). Test of significance (C). Standardized Canonical Discriminant Function Coefficients (D). Classification Results of Discriminant Analysis (E). (DOCX) [file pone.0152907.s019.docx]

**S7 Table**. Variables Entered/Removed in the stepwise discriminant analysis of tomato shape **(A).** Summary of canonical discriminant functions- Eigenvalues **(B).** Test of significance **(C).** Standardized Canonical Discriminant Function Coefficients **(D).** Classification Results of Discriminant Analysis **(E).**

**A**

|  |  | **Wilks' Lambda** | |  |  |  |  |  |  |
| --- | --- | --- | --- | --- | --- | --- | --- | --- | --- |
|  |  |  |  |  |  | **Exact F** |  |  |  |
|  | **Step Entered** | **Statistic** | **df1** | **df2** | **df3** | **Statistic** | **df1** | **df2** | **Sig.** |
| 1 | Fruit Shape Index External.1 | 0.41 | 1 | 1 | 108 | 155.194 | 1 | 108 | 0 |
| 2 | Height Mid-width | 0.352 | 2 | 1 | 108 | 98.295 | 2 | 107 | 0 |
| 3 | Circular | 0.323 | 3 | 1 | 108 | 74.082 | 3 | 106 | 0 |
| 4 | Lobedness Degree | 0.261 | 4 | 1 | 108 | 74.379 | 4 | 105 | 0 |
| 5 | H. Asymmetry.ov | 0.245 | 5 | 1 | 108 | 63.981 | 5 | 104 | 0 |
| 6 | Proximal Indentation Area | 0.235 | 6 | 1 | 108 | 55.732 | 6 | 103 | 0 |

At each step, the variable that minimizes the overall Wilks' Lambda is entered.

**B**

| **Function** | **Eigenvalue** | | | **% of Variance** | | **Cumulative %** |  | **Canonical Correlation** | | |
| --- | --- | --- | --- | --- | --- | --- | --- | --- | --- | --- |
| DF1 |  | 3.247 |  | 100 | | 100 |  |  | 0.874 | |
| **C** | | | | | |  |  |  |  |  |
|  | | |  |  |  |  |  |  |  |  |
| **Test of Function(s)** | | |  | **Wilks' Lambda** | | **Chi-square** |  | **df** |  | **Sig.** |
| DF1 |  |  |  | 0.235 | | 151.841 | 6 | |  | 0 |
| **D** | | | | | | | | |  |  |
|  |  |  |  |  |  |  |  | **Function Df1** | | |
| Height Mid-width | | | |  |  |  |  |  | 0.529 | |
| Fruit Shape Index External.1 | | | | | |  |  |  | -1.08 | |
| Circular |  |  |  |  |  |  |  |  | 2.459 | |
| Proximal Indentation Area | | | |  |  |  |  |  | -0.355 | |
| H. Asymmetry.ov | | | |  |  |  |  |  | -0.391 | |
| Lobedness Degree | | | |  |  |  |  |  | 2.42 | |
| **E** | | | | | | |  |  |  |  |
|  |  |  |  |  |  |  | | |  |  |
|  |  |  |  |  |  | **Predicted Group Membership** | | |  | **Total** |
|  |  |  |  |  | Shape | Round |  | Flat |  |  |
| Original |  | % | |  | Round | 100 | 0 | | 100 | |
|  |  |  |  |  | Flat | 0 | 100 | | 100 | |
| Cross-validated | | % | |  | Round | 97.6 | 2.4 | | 100 | |
|  |  |  |  |  | Flat | 0 | 100 | | 100 | |

100.0% of original grouped cases correctly classified.

99.1% of cross-validated grouped cases correctly classified.
