# Supplementary material for: Tomato Fruits Show Wide Phenomic Diversity but Fruit Developmental Genes Show Low Genomic Diversity
Source: PLoS One. 2016 Apr 14;11(4):e0152907. doi: 10.1371/journal.pone.0152907 (PMC4831840; doi:10.1371/journal.pone.0152907)
Supplement: S8 Table — (DOCX) [file pone.0152907.s020.docx]

**S8 Table.** Pearson’s Correlation between fruit parameters.

| Variables | **Area** | **FW** | **FSIE.1** | **DEFB** | **FST** | **Ellipsoid** | **Rect** | **PIA** | **DIA** | **Cir** | **Obovoid** | **Ovoid** | **V. Aym** | **D Ecc** | **EccAI** | **PA** | **PT** | **Lum** | **L*** | **a*** | **b*** | **Hue** | **Chroma** | **TC** | **°Brix** | **pH** |
| --- | --- | --- | --- | --- | --- | --- | --- | --- | --- | --- | --- | --- | --- | --- | --- | --- | --- | --- | --- | --- | --- | --- | --- | --- | --- | --- |
| **Area** | **1** |  |  |  |  |  |  |  |  |  |  |  |  |  |  |  |  |  |  |  |  |  |  |  |  |  |
| **FW** | **0.763** | **1** |  |  |  |  |  |  |  |  |  |  |  |  |  |  |  |  |  |  |  |  |  |  |  |  |
| **FSIE.1** | -0.047 | -0.096 | **1** |  |  |  |  |  |  |  |  |  |  |  |  |  |  |  |  |  |  |  |  |  |  |  |
| **DEFB** | 0.039 | 0.013 | -0.054 | **1** |  |  |  |  |  |  |  |  |  |  |  |  |  |  |  |  |  |  |  |  |  |  |
| **FST** | 0.164 | 0.140 | **-0.181** | **-0.727** | **1** |  |  |  |  |  |  |  |  |  |  |  |  |  |  |  |  |  |  |  |  |  |
| **Ellipsoid** | **-0.322** | **-0.294** | **0.394** | **-0.257** | -0.130 | **1** |  |  |  |  |  |  |  |  |  |  |  |  |  |  |  |  |  |  |  |  |
| **Rect** | 0.056 | -0.011 | **-0.350** | 0.174 | 0.078 | **-0.221** | **1** |  |  |  |  |  |  |  |  |  |  |  |  |  |  |  |  |  |  |  |
| **PIA** | **0.340** | **0.336** | **-0.521** | 0.088 | **0.267** | **-0.811** | **0.420** | **1** |  |  |  |  |  |  |  |  |  |  |  |  |  |  |  |  |  |  |
| **DIA** | -0.002 | -0.024 | **-0.274** | **0.340** | -0.141 | **-0.407** | **0.249** | **0.252** | **1** |  |  |  |  |  |  |  |  |  |  |  |  |  |  |  |  |  |
| **Cir** | **-0.245** | -0.164 | **0.358** | **-0.419** | 0.034 | **0.737** | **-0.448** | **-0.629** | **-0.529** | **1** |  |  |  |  |  |  |  |  |  |  |  |  |  |  |  |  |
| **Obovoid** | -0.033 | -0.087 | **0.309** | **0.283** | **-0.312** | 0.047 | -0.052 | -0.088 | -0.068 | -0.157 | **1** |  |  |  |  |  |  |  |  |  |  |  |  |  |  |  |
| **Ovoid** | 0.085 | 0.119 | **-0.217** | **-0.217** | **0.338** | **-0.185** | 0.024 | 0.127 | 0.095 | 0.112 | **-0.748** | **1** |  |  |  |  |  |  |  |  |  |  |  |  |  |  |
| **V. Asym** | **0.528** | **0.422** | -0.169 | **0.272** | -0.033 | **-0.575** | **-0.251** | **0.282** | **0.281** | **-0.427** | 0.046 | 0.009 | **1** |  |  |  |  |  |  |  |  |  |  |  |  |  |
| **D Ecc** | **-0.216** | -0.168 | **-0.231** | 0.028 | -0.131 | **0.210** | **0.290** | 0.009 | 0.156 | 0.042 | -0.066 | -0.112 | **-0.277** | **1** |  |  |  |  |  |  |  |  |  |  |  |  |
| **EccAI** | **0.419** | **0.312** | -0.105 | 0.084 | **0.217** | **-0.518** | 0.087 | **0.384** | 0.111 | **-0.374** | -0.043 | 0.169 | **0.422** | **-0.768** | **1** |  |  |  |  |  |  |  |  |  |  |  |
| **PA** | -0.003 | -0.047 | **0.356** | 0.083 | **-0.182** | 0.130 | -0.024 | **-0.256** | 0.126 | 0.125 | 0.026 | 0.125 | 0.014 | -0.166 | 0.046 | **1** |  |  |  |  |  |  |  |  |  |  |
| **PT** | -0.078 | -0.094 | **0.269** | 0.057 | **-0.217** | **0.245** | -0.033 | **-0.340** | 0.089 | **0.224** | 0.038 | 0.037 | -0.063 | -0.030 | -0.104 | **0.710** | **1** |  |  |  |  |  |  |  |  |  |
| **Lum** | **0.312** | **0.246** | -0.048 | 0.121 | 0.116 | **-0.246** | 0.072 | **0.284** | 0.100 | **-0.313** | -0.009 | 0.016 | **0.225** | -0.111 | **0.243** | -0.143 | **-0.183** | **1** |  |  |  |  |  |  |  |  |
| **L*** | 0.078 | 0.084 | -0.065 | 0.048 | 0.106 | -0.125 | 0.014 | 0.163 | 0.026 | -0.154 | -0.026 | 0.060 | 0.086 | -0.029 | 0.102 | -0.166 | **-0.179** | **0.818** | **1** |  |  |  |  |  |  |  |
| **a*** | 0.117 | 0.067 | -0.010 | 0.063 | 0.012 | -0.077 | 0.063 | 0.080 | 0.132 | -0.113 | -0.071 | 0.016 | 0.052 | -0.060 | 0.081 | 0.055 | 0.023 | -0.092 | **-0.587** | **1** |  |  |  |  |  |  |
| **b*** | **-0.370** | **-0.248** | -0.129 | -0.055 | 0.091 | 0.071 | -0.029 | -0.023 | 0.012 | 0.093 | -0.158 | **0.183** | **-0.204** | 0.116 | -0.151 | -0.145 | -0.130 | **0.225** | **0.653** | **-0.514** | **1** |  |  |  |  |  |
| **Hue** | **-0.203** | -0.127 | -0.030 | -0.065 | -0.009 | 0.072 | -0.047 | -0.062 | -0.104 | 0.118 | 0.064 | 0.005 | -0.093 | 0.113 | -0.125 | -0.071 | -0.034 | 0.110 | **0.624** | **-0.975** | **0.629** | **1** |  |  |  |  |
| **Chroma** | **-0.267** | -0.172 | -0.121 | -0.052 | 0.128 | 0.045 | -0.044 | -0.007 | 0.082 | 0.041 | **-0.222** | **0.236** | -0.162 | 0.056 | -0.106 | -0.119 | -0.131 | **0.237** | **0.491** | -0.129 | **0.883** | **0.246** | **1** |  |  |  |
| **TC** | -0.067 | -0.119 | 0.049 | -0.174 | **0.196** | 0.061 | 0.137 | -0.050 | 0.120 | -0.069 | -0.111 | 0.079 | -0.113 | 0.004 | 0.018 | 0.070 | 0.112 | -0.050 | **-0.246** | **0.465** | -0.128 | **-0.430** | 0.060 | **1** |  |  |
| **°Brix** | -0.173 | -0.135 | -0.105 | **-0.299** | **0.315** | 0.118 | **0.220** | 0.108 | -0.125 | 0.027 | -0.076 | -0.042 | **-0.304** | 0.039 | -0.013 | **-0.204** | -0.141 | 0.078 | 0.028 | 0.135 | 0.073 | -0.111 | 0.150 | **0.254** | **1** |  |
| **pH** | -0.090 | -0.054 | 0.000 | **0.181** | **-0.216** | 0.049 | -0.021 | -0.085 | -0.051 | 0.072 | 0.078 | -0.078 | -0.027 | 0.065 | -0.073 | 0.071 | 0.095 | **-0.195** | -0.152 | -0.024 | -0.063 | 0.047 | -0.114 | -0.105 | -0.142 | **1** |

*Values in bold are different from 0 with a significance level alpha=0.05*

**FW:** Fruit Weight; **FSIE.1**: Fruit Shape Index External.1; **DEFB**:Distal End Fruit Blockiness; **FST**: Fruit Shape Triangle; **Rect**: Rectangular; PIA: Proximal Indentation Area; **DIA**: Distal Indentation Area; **Cir**: Circular; **V. Asym**: Vertical Asymmetry; **D Ecc**: Distal Eccentricity; **EccAI**: Eccentricity Area Index; **PA**: Pericarp Area; **PT**: Pericarp Thickness; **Lum**: Avg. Luminosity; **TC**: Total Carotenoid
