# Supplementary material for: Tomato Fruits Show Wide Phenomic Diversity but Fruit Developmental Genes Show Low Genomic Diversity
Source: PLoS One. 2016 Apr 14;11(4):e0152907. doi: 10.1371/journal.pone.0152907 (PMC4831840; doi:10.1371/journal.pone.0152907)
Supplement: S9 Table — (DOCX) [file pone.0152907.s021.docx]

**S9 Table.** Univariate statistical analysis for selected fruit parameters of 127 accessions.

|  | **Min** | **Max** | **Mean** | **SD** | **CV** | **Mean+SD** | **Mean-SD** | **Mean+2SD** | **Mean-2SD** | **No. of accessions (%)** | | | | |
| --- | --- | --- | --- | --- | --- | --- | --- | --- | --- | --- | --- | --- | --- | --- |
|  |  |  |  |  |  |  |  |  |  | **Highly**  **Undesirable**  **(<mean -2SD)** | **Undesirable**  **(<mean -SD)** | **Average**  **(between**  **mean±SD)** | **Desirable**  **(>mean+SD)** | **Highly desirable (>mean+2SD)** |
| **Fruit weight** | 1.00 | 334.00 | 37.39 | 44.86 | 1.20 | 82.25 | -7.47 | 127.11 | -52.33 | 0.00 | 0 | 116(91.33%) | 6(4.72%) | 5(3.93%) |
| **VD/HD** | 0.58 | 1.48 | 0.97 | 0.18 | 0.18 | 1.16 | 0.79 | 1.34 | 0.61 | 2(1.57%)^a^ | 20(15.74%)^a^ | 86(67.71%)^b^ | 14(11.02%)^c^ | 5(3.93%)^c^ |
| **a*/b*** | -0.37 | 0.90 | 0.28 | 0.23 | 0.81 | 0.52 | 0.05 | 0.75 | -0.18 | 6(4.72%) | 13(10.23%) | 92(72.44%) | 14(11.02%) | 2(1.57%) |
| **Total carotenoid** | 11.71 | 177.72 | 66.08 | 32.96 | 0.50 | 99.04 | 33.12 | 132.00 | 0.17 | 0 | 14(11.02%) | 93(73.23%) | 20(15.75%) | 7(5.51%) |
| **°Brix** | 1.80 | 7.50 | 4.38 | 1.28 | 0.29 | 5.66 | 3.10 | 6.95 | 1.81 | 1(0.78%) | 20(15.74%) | 74(58.26%) | 30(23.62%) | 2(1.57%) |

**^a^**: denotes squat shape; **^b^:** denotes circular shape; **^c^**: denotes elongated shape
