# Supplementary material for: Tomato Fruits Show Wide Phenomic Diversity but Fruit Developmental Genes Show Low Genomic Diversity
Source: PLoS One. 2016 Apr 14;11(4):e0152907. doi: 10.1371/journal.pone.0152907 (PMC4831840; doi:10.1371/journal.pone.0152907)
Supplement: S10 Table — (DOCX) [file pone.0152907.s022.docx]

**S10 Table**. Annotation of the genes selected for Eco TILLING

| **Gene** | **Gene abbreviation** | | **Arabidopsis gene i.d.** | **Tomato gene i.d.** | **Tomato chromosome** | **Pathway compartment** | **Arabidopsis annotation** | **Gene reference** |
| --- | --- | --- | --- | --- | --- | --- | --- | --- |
| 1-aminocyclopropane-1carboxylate synthase 2 | *ACS2* | | AT1G01480.1 | Solyc01g095080.2.1 | SL2.40ch01 | Ethylene biosynthesis | Pyridoxal phosphate dependent transferase | Yip et al. 1992 |
| CONSTITUTIVE PHOTOMORPHOGENIC 1 homolog | | *COP1 HOMOLOG* | AT2G32950.1 | Solyc12g005950.1.1 | SL2.40ch12 | Photomorphogenesis | E3 ubiquitin ligase | OrdoñezHerrera et al. 2014 |
|  |  | |  |  |  |  |  |  |
| Lycopene beta cyclase | *CYC-B* | | AT3G10230.1 | Solyc06g074240.1.1 | SL2.40ch06 | Carotenoid biosynthesis | Lycopene cyclase-type, FAD-binding | Ronen et al. 2000 |
| DNA mismatch repair homolog MSH2 | *MSH2* | | AT3G18524.1 | Solyc06g069230.2.1 | SL2.40ch06 | DNA repair | DNA mismatch repair protein | Gómez and Spampinato 2013 |
| NAC domain protein  non-ripening | *NAC-NOR* | | AT3G15510.1 | Solyc10g006880.2.1 | SL2.40ch10 | DNA binding transcriptional factor | No apical meristem (NAM) proteins | Kunieda et al. 2008 |
| Phototropin1 | *PHOT1* | | AT3G45780.1 | Solyc11g072710.1.1 | SL2.40ch11 | Photoreception | Protein kinase like domain | Sharma et al. 2014 |
| Phytochrome A | *PHYA* | | 1G09570.1 | Solyc10g044670.1.1 | SL2.40ch10 | Photoreception | Histidine kinase | Weller et al. 2000 |
| Phytochrome B1 | *PHYB1* | | AT2G18790.1 | Solyc01g059870.2.1 | SL2.40ch01 | Photoreception | Histidine kinase | Weller et al. 2001 |
| Phytoene synthase 1 | *PSY1* | | AT5G17230.3 | Solyc03g031860.2.1 | SL2.40ch03 | Carotenoid biosynthesis | Squalene/phytoene synthase | Giorio et al. 2008 |
| Ripening inhibitor /macrocalyx | *RIN/MC* | | AT2G03710.1 | Solyc05g056620.1.1 | SL2.40ch05 | MADS box Transcriptional factor | MADS box transcriptional factor | Vrebalov et al. 2002 |

**References**

Giorio G, Stigliani AL, D’Ambrosio C (2008) Phytoene synthase genes in tomato (Solanum lycopersicum L.)–new data on the structures, the deduced amino acid sequences and the expression patterns. FEBS journal 275:527-535

Gómez R, Spampinato CP (2013) Mismatch recognition function of Arabidopsis thaliana MutSγ. DNA repair 12:257-264

Kunieda T et al. (2008) NAC family proteins NARS1/NAC2 and NARS2/NAM in the outer integument regulate embryogenesis in Arabidopsis. The Plant Cell Online 20:2631-2642

Ordoñez-Herrera N, Fackendahl P, Yu X, Schaefer S, Koncz C, Hoecker U (2015) A cop1/spa mutant deficient in COP1 and SPA proteins reveals partial coaction of COP1 and SPA during Arabidopsis post-embryonic development and photomorphogenesis. Molecular Plant 8, 479–481

Ronen G, Carmel-Goren L, Zamir DH J (2000) An alternative pathway to betacarotene formation in plant chromoplasts discovered by map-based cloning of Beta and old-gold color mutations in tomato. Proceedings of the National Academy of Sciences of the United States of America 97:11102- 11107

Sharma S, Kharshiing E, Srinivas A, Zikihara K, Tokutomi S, Nagatani A, Fukayama H, Bodanapu R, Behera RK, Sreelakshmi Y, Sharma R (2014) A Dominant Mutation in LOV2 Domain Vicinity Impairs Phototropin1 Signaling in Tomato. Plant Physiol 164: 2030-2044

Vrebalov J et al. (2002) A MADS-box gene necessary for fruit ripening at the tomato ripening-inhibitor (rin) locus. Science 296:343-346

Weller JL, Perrotta G, Schreuder ME, Van Tuinen A, Koornneef M, Giuliano G, Kendrick RE (2001) Genetic dissection of blue‐light sensing in tomato using mutants deficient in cryptochrome 1 and phytochromes A, B1 and B2. The Plant Journal 25:427-440

Weller JL, Schreuder ME, Smith H, Koornneef M, Kendrick RE (2000) Physiological interactions of phytochromes A, B1 and B2 in the control of development in tomato. The Plant Journal 24:345- 356

Yip W-K, Moore T, Yang SF (1992) Differential accumulation of transcripts for four tomato 1- aminocyclopropane-1-carboxylate synthase homologs under various conditions. Proceedings of the National Academy of Sciences. 89:2475-2479
