# Supplementary material for: Tomato Fruits Show Wide Phenomic Diversity but Fruit Developmental Genes Show Low Genomic Diversity
Source: PLoS One. 2016 Apr 14;11(4):e0152907. doi: 10.1371/journal.pone.0152907 (PMC4831840; doi:10.1371/journal.pone.0152907)
Supplement: S11 Table — (DOCX) [file pone.0152907.s023.docx]

**S11 Table.** Nature of nucleotide changes and its proportion.

| **Nature of change** | **Nucleotide** | **Number** | **Proportion** |
| --- | --- | --- | --- |
|  | **Change** |  |  |
| **Transition** | G:A | 11 | 0.2037 |
|  | A:G | 6 | 0.11111 |
|  | C:T | 4 | 0.07407 |
|  | T:C | 2 | 0.03704 |
|  | Sub total | 23 | 0.42592 |
| **Transversion** | A:T | 4 | 0.07407 |
|  | G:T | 4 | 0.07407 |
|  | A:C | 3 | 0.05556 |
|  | T:A | 3 | 0.05556 |
|  | T:G | 3 | 0.05556 |
|  | G:C | 2 | 0.03704 |
|  | C:A | 1 | 0.01852 |
|  | Sub total | 20 | 0.37038 |
| **Indels** | A:- | 4 | 0.07407 |
|  | C:- | 1 | 0.01852 |
|  | -:A | 3 | 0.05556 |
|  | -:C | 2 | 0.03704 |
|  | -:G | 1 | 0.01852 |
|  | Sub total | 11 | 0.20371 |
|  | Total | 54 | 1.00001 |
