# Supplementary material for: Tomato Fruits Show Wide Phenomic Diversity but Fruit Developmental Genes Show Low Genomic Diversity
Source: PLoS One. 2016 Apr 14;11(4):e0152907. doi: 10.1371/journal.pone.0152907 (PMC4831840; doi:10.1371/journal.pone.0152907)
Supplement: S12 Table — (DOCX) [file pone.0152907.s024.docx]

**S12 Table.** Neutrality tests.

| **Gene** | **Overall** | **Coding region** |  |
| --- | --- | --- | --- |
|  | **Tajima’s D** | **Tajima’s D** | **Ka/Ks** |
|  |  | **(Nonsyn/Syn)** |  |
| ***ACS2*** | **-2.13724*** (P<0.05) | n.d. | n.d. |
| ***COP1*** | -1.16088 | n.d. | n.d. |
| ***CYC-B*** | -1.12618 | n.d. | n.d. |
| ***MSH2*** | -1.34601 | n.d. | n.d. |
| ***NAC-NOR*** | -1.10488 | n.d. | n.d. |
| ***PHOT1*** | **-2.30944****(P<0.01) | n.d. | n.d. |
| ***PHYA*** | -1.57432 | 1.344 | 0.575 |
| ***PHYB1*** | -1.57432 | n.d. | n.d. |
| ***PSY1*** | NA | n.d. | n.d. |
| ***RIN*** | NA | n.d. | n.d. |

*n.d.- Not detected*
